# Robustness from flexibility in the fungal circadian clock

**DOI:** 10.1186/1752-0509-4-88

**Published:** 2010-06-24

**Authors:** Ozgur E Akman, David A Rand, Paul E Brown, Andrew J Millar

**Affiliations:** 1 Centre for Systems Biology at Edinburgh, The University of Edinburgh, Edinburgh, UK; 2 Interdisciplinary Programme for Cellular Regulation, University of Warwick, Coventry, UK; 3 Systems Biology Centre, University of Warwick, Coventry, UK; 4 School of Biological Sciences, University of Edinburgh, Edinburgh, UK; 5 School of Engineering, Computing & Mathematics, University of Exeter, Exeter, UK

## Abstract

**Background:**

Robustness is a central property of living systems, enabling function to be maintained against environmental perturbations. A key challenge is to identify the structures in biological circuits that confer system-level properties such as robustness. Circadian clocks allow organisms to adapt to the predictable changes of the 24-hour day/night cycle by generating endogenous rhythms that can be entrained to the external cycle. In all organisms, the clock circuits typically comprise multiple interlocked feedback loops controlling the rhythmic expression of key genes. Previously, we showed that such architectures increase the flexibility of the clock's rhythmic behaviour. We now test the relationship between flexibility and robustness, using a mathematical model of the circuit controlling conidiation in the fungus *Neurospora crassa*.

**Results:**

The circuit modelled in this work consists of a central negative feedback loop, in which the *frequency *(*frq*) gene inhibits its transcriptional activator *white collar-1 *(*wc-1*), interlocked with a positive feedback loop in which FRQ protein upregulates WC-1 production. Importantly, our model reproduces the observed entrainment of this circuit under light/dark cycles with varying photoperiod and cycle duration. Our simulations show that whilst the level of *frq *mRNA is driven directly by the light input, the falling phase of FRQ protein, a molecular correlate of conidiation, maintains a constant phase that is uncoupled from the times of dawn and dusk. The model predicts the behaviour of mutants that uncouple WC-1 production from FRQ's positive feedback, and shows that the positive loop enhances the buffering of conidiation phase against seasonal photoperiod changes. This property is quantified using Kitano's measure for the overall robustness of a regulated system output. Further analysis demonstrates that this functional robustness is a consequence of the greater evolutionary flexibility conferred on the circuit by the interlocking loop structure.

**Conclusions:**

Our model shows that the behaviour of the fungal clock in light-dark cycles can be accounted for by a transcription-translation feedback model of the central FRQ-WC oscillator. More generally, we provide an example of a biological circuit in which greater flexibility yields improved robustness, while also introducing novel sensitivity analysis techniques applicable to a broader range of cellular oscillators.

## Background

A circadian network (or biological clock) confers a competitive advantage to an organism, probably by enabling it to anticipate cyclic changes in the environment. Circadian rhythms with very similar properties are found in almost all organisms, controlling processes from cyanobacterial cell division to human sleep-wake cycles [1]. There is now evidence that these rhythms can be generated by loops of genes and gene products that communicate by positive and negative feedback. Such loops have been experimentally elucidated for a variety of organisms, including the fungus *Neurospora crassa*, the fly *Drosophila melanogaster *and the plant *Arabidopsis thaliana *[2]. Input signals from light and/or temperature alter the level of one or more components of the loops in order to reset the phase of the rhythm [2].

For the circadian clock to provide an adaptive advantage, it is important for it to maintain the appropriate phase relationship relative to dawn and dusk such that rhythmic biological processes occur at the optimal time of the day. The responses of the clock must ensure that this phase relationship changes appropriately when the clock is subject to regular perturbations - such as seasonal changes in photoperiod and temperature - while being resilient to the more or less random perturbations resulting from evolutionary processes, external environmental fluctuations or due to the stochastic environment of the cell. The existence of these experimentally tractable system outputs and related performance measures, together with increasingly detailed genetic information, complex dynamics and easy manipulation by light and temperature signals means that circadian clocks are good systems for investigating how the structures of signalling networks affect their system-level properties.

In this vein, recent theoretical and experimental work has focused on elucidating the relationships between the multi-loop architectures characteristic of circadian systems, the flexibility of the clock's dynamic behaviour and the robustness of its function in biological timing [3-9]. Flexibility measures how readily the rhythmic profiles of all the molecular clock components can be altered by modifying the biochemical parameters or environmental inputs of the clock circuit [3]. Robustness focuses on how a biological function, such as the phase of a particular clock component, is maintained under varying conditions. The relationship between these two high-level properties is a complex one, depending on the particular properties of the system of interest. Although flexibility may decrease robustness by increasing sensitivity to perturbations, it can also yield greater robustness by enhancing the ability of the network to tune key environmental responses [10]. Studies within specific circadian systems have had success in identifying the components and structures contributing to their robustness [4,6]. In a more general context, Kitano recently proposed a simple, scalar measure of robustness that aimed to facilitate comparisons across widely differing biological systems [11]. Here, we combine these complementary approaches to analyse the fungal circadian clock.

### The Neurospora circadian clock

The fungus *Neurospora crassa *has one of the most comprehensively studied and best understood circadian systems [12,13]. *Neurospora *exhibits a 22 hour rhythm in asexual spore formation (conidiation) when grown in constant darkness (DD) as well as circadian rhythms in metabolism, stress response and other physiological processes [14]. The conidiation rhythm can be entrained by both light and temperature cycles, exhibiting either *systematic *or *driven *entrainment depending on the forcing protocol used [15]. In 24 hr light-dark (LD) cycles, the phase of entrainment (judged by the time of conidiation onset) coincides with the middle of the night in both long and short days [16]. The phase of the clock thus varies systematically with photoperiod: both dusk and dawn signals are integrated to set phase rather than phase being determined solely by either signal alone [15]. By contrast, the clock exhibits driven entrainment in symmetric photic T-cycles (LD cycles of different lengths *T *with 50% of the cycle in light and 50% in dark). Under these conditions, conidiation onset occurs a fixed time (≈ 7 hrs) after dusk irrespective of cycle length [17].

The core multi-loop genetic oscillator believed to underlie many of the observed circadian rhythms in *Neurospora *- including the conidiation rhythm - is formed by the rhythmic gene *frequency (frq*), and the constitutively expressed genes *white collar-1 (wc-1) *and *white collar-2 (wc-2) *[13]. The protein products of the *white collar *genes, WC-1 and WC-2, comprise the positive elements of a central negative feedback loop. WC-1 and WC-2 form a heterodimeric WHITE COLLAR complex (WCC) which binds to two light-response elements (LREs) in the *frq *promoter, activating transcription of *frq *[18,19]. The protein product of the *frq *gene is the negative element of the loop. Following transcription of *frq*, two isoforms of FRQ protein are expressed and form homodimeric complexes [20,21]. The relative levels of these isoforms changes with temperature as a result of thermosensitive splicing, yielding a bifurcated, temperature-dependent protein pathway [22,23]. When the expression of the FRQ isoforms reaches a certain level, they interact with the WCC to inhibit its activation of *frq *transcription, closing the negative feedback loop [18,24-28]. The inhibition of *frq *transcription appears to be the consequence of FRQ binding to the WCC and clearing it from the nucleus [28]. The FRQ-WCC interaction is mediated by the protein product of an RNA helicase (*frh*) [29].

As well as forming the negative element of the loop, FRQ positively regulates expression of WC-1, giving a positive feedback loop that interlocks with the primary loop [18,30]. In addition to its essential role in the *Neurospora *feedback loops, WC-1 is a blue-light photoreceptor necessary for photoentrainment. Blue light is perceived by a flavin chromophore (FAD) that binds to the LOV domain of WC-1. The corresponding WCC bound to the LREs at the *frq *promoter is a slower migrating complex than that present in the dark. Light appears to reset the clock by causing an increase in the relative concentration of the slower complex [19], resulting in enhanced transcription of *frq *[31].

In constant darkness, *frq *mRNA is at a minimum level early in the subjective night, peaking early in the subjective day. FRQ peaks 4-6 hrs after its transcript, reaching minimum levels approximately 12 hrs later. *wc-1 *mRNA is expressed constitutively, with its protein product oscillating roughly in antiphase with FRQ [19,24-26,30]. In constant light, levels of both *frq *mRNA and FRQ are elevated and arrhythmic, indicating that the central FREQUENCY-WHITE COLLAR (FRQ-WC) clock is not functioning [31,32]. In 24 hr LD cycles, acute light responses give rise to *frq *mRNA profiles that directly reflect the light environment in different photoperiods; by contrast, the FRQ protein profile appears to determine the onset of conidiation [16].

### Modelling the clock

The discovery of the molecular machinery underlying the *Neurospora *circadian network has led to the development of a number of mathematical models of the clock [8,33-39]. These have enabled a range of issues to be addressed regarding the functional relationship between the architecture of the clock and the maintenance of circadian function, including the mechanisms underlying the buffering of free-running period and amplitude against seasonal temperature variations and molecular noise. Thus far, the models developed have primarily concentrated on the expression of the core clock genes in free-running conditions (constant darkness), with the effect of light modelled through direct changes to transcription and degradation rates. Such models therefore have limited use in analysing the photoperiodic responses of the clock.

In this work, we present a model based on the core FRQ-WC oscillator that incorporates both the negative *frq *and positive *wc-1 *loops, as well as part of the light-signalling pathway. In addition to simulating the behaviour of the clock in constant conditions (DD), we show that this increased level of biological detail enables our model to reproduce the experimentally observed disassociation between light-driven *frq *mRNA and photoperiodic FRQ protein in 24 hr LD cycles, as well as the driven behaviour seen in symmetric LD T-cycles. This suggests that at least some of the entrainment properties of the *Neurospora *clock can be accounted for by a transcription-translation feedback model of the FRQ-WC oscillator. By using our model to simulate the effect of decoupling the positive *wc-1 *loop from the negative *frq *loop, we predict that one of the possible benefits conferred by the presence of the positive loop is robustness of entrained phase against seasonal variations in photoperiod. This yields the experimentally testable prediction that decoupling the loops will result in a dusk-driven clock in long days. The decoupling simulations also provide an additional testable prediction regarding the specific dynamical mechanism underlying the loss of free-running rhythmicity observed in experimental *Neurospora *strains lacking the *wc-1 *loop.

We also introduce a simple measure of the flexibility of the network based on quantifying how outputs of the entrained clock vary under parameter perturbations achievable by evolutionary processes [3,40]. Using this measure, we demonstrate that the positive loop yields a more flexible clock. This increased flexibility is shown to be primarily characterised by a greater flexibility in entrained phase, leading to the enhanced robustness against photoperiod fluctuations suggested by the phase simulations. Our results thus provide an example of a cellular circuit where improved robustness is linked directly to increased flexibility.

## Results

### Description of the model

**Figure 1:**
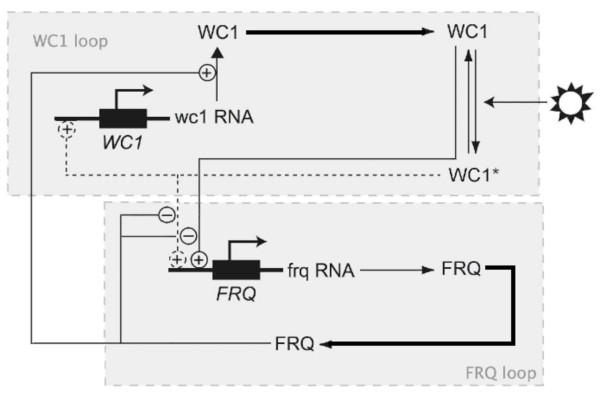
**Network diagram for the mathematical model of the Neurospora clock**. The model incorporates the core genes *frequency *(*frq*) and *white collar-1 *(*wc-1 *). The protein product of the *wc-1 *gene (WC-1) is the positive element of a central negative feedback loop, while the *frq *protein product (FRQ) is the negative element. FRQ also upregulates the level of WC-1 yielding a positive feedback loop interlocked with the primary one. WC1* represents light-activated WC-1. Thicker lines denote the delay between the translation of a protein and conversion into its active form, modelled using a distributed delay.

A network representation of our model of the *Neurospora *clock is shown in Figure 1. The model comprises a set of five coupled ordinary integrodifferential equations describing the dynamics of the two core circadian genes *frq *and *wc-1*. It does not include the genes *wc-2 *and *frh *as their protein products form complexes with WC-1 and FRQ respectively and can therefore be combined with these proteins without fundamentally modifying the resulting model. For simplicity, the model in its current form also does not include the light-responsive clock gene *vivid *(*vvd*), a key repressor of light-induced expression controlled by the WCC [41-43], which is believed to sustain a clock that runs during the day [44]. Finally, since we do not consider temperature responses here, we do not differentiate between the two different FRQ isoforms.

### Simulations of mRNA and protein profiles

**Figure 2:**
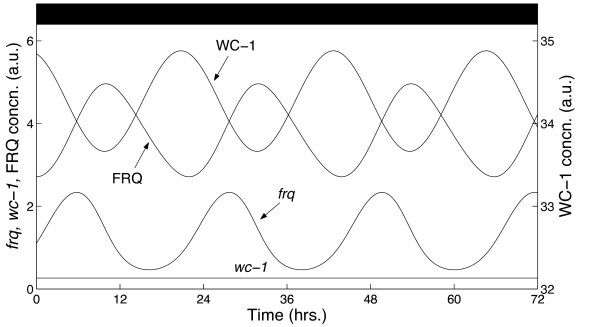
**Simulated mRNA and protein profiles in DD**. The time series qualitatively match experimental data, yielding: i) an oscillation period close to 22 hrs; ii) constant *wc-1 *levels; and iii) a FRQ profile which oscillates in antiphase with WC-1, reaching peak levels shortly after its transcript [19,24-26,30].

**Figure 3:**
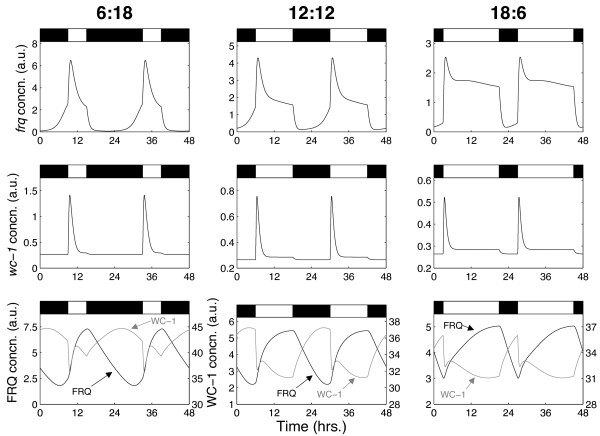
**Simulations of the model in different photoperiods**. Both *frq *and *wc-1 *mRNA exhibit rapid increases in expression at lights-on, while *frq *mRNA also exhibits a rapid decrease in expression at lights-off, consistent with experimental data [16]. The model also reproduces the convergence of *frq *and *wc-1 *to equilibrium levels following dawn in longer photoperiods.

Figures 2 and 3 show simulations of the model in DD and LD conditions respectively. The parameter values used to generate these solutions were obtained by minimising a cost function quantifying the goodness-of-fit of the model to experimental time courses [45-48]. This measured how well simulated solutions matched certain key features of the data, such as the free-running period of the clock and the peak and trough phases of the clock components in both free-running and entrained conditions [46]. The DD simulation has a period close to the observed value of 22 hrs, with relative phase relationships also consistent with experimental data: the delay between the peaks of *frq *transcript and FRQ protein is approximately 5 hrs, while FRQ and WC-1 protein oscillate roughly in antiphase.

Furthermore, despite the fact the cost function only assesses goodness-of-fit in simulated 12:12 LD cycles, the optimal solution is a good match to data in long and short days also. As reported experimentally, in all photoperiods for which the clock is stably entrained, *frq *and *wc-1 *transcripts exhibit rapid induction at dawn while *frq *expression falls rapidly at dusk, with both transcripts converging to an equilibrium level during the light phase in long days [16]. By contrast, FRQ protein displays markedly smoother changes in expression level, increasing slowly from a minimum level around dawn to a peak level around dusk before degrading back down to its minimum at a roughly constant rate.

### Simulations of conidiation onset

Experimental work has suggested a correlation between the FRQ protein profile and the phase of the visible rhythm of conidiation onset [15,16]. Specifically, it has been proposed that conidiation phase coincides with a fall in FRQ expression to a point roughly halfway between its maximum and minimum values, possibly as a result of the derepression of a clock output pathway controlling conidia formation. This is based on the observation that in 24 hr LD cycles the decrease in FRQ to the peak-trough midpoint is attained approximately at midnight across photoperiods, coincident with the time at which conidial spores begin to be formed [16].

**Figure 4:**
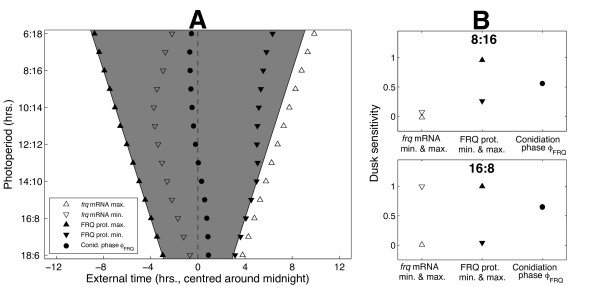
**The model reproduces the systematic entrainment observed in LD cycles**. **A**. Simulated variation of conidiation onset with photoperiod length. As in [16], conidiation onset was identified with the time *ϕ_FRQ _*at which FRQ has decreased to the approximate midpoint of its peak and trough values. Peak and trough times of *frq *mRNA and FRQ protein are also shown. White and grey regions denote light and dark respectively while the dotted line indicates the middle of the night. **B**. Dusk sensitivities ∂*ϕ/*∂*t_DUSK _*of the phase measures plotted in A for short and long days (see Additional file 1, Figure S2A for the sensitivities at intermediate photoperiods). The peak and trough times of *frq *mRNA are locked to either dusk (∂*ϕ/*∂*t_DUSK _*= 1) or dawn (∂*ϕ/*∂*t_DUSK _*= 0). By contrast, conidiation onset *ϕ_FRQ _*varies systematically with photoperiod (∂*ϕ_FRQ_/*∂*t_DUSK _≈ *0.5).

Figure 4A shows how the simulated phase *ϕ_FRQ_* of this molecular conidiation correlate varies with photoperiod. It can be seen that the peak of *frq *mRNA expression is locked to dawn, while the trough is locked to dawn in short days and dusk in all other photoperiods. Conidiation phase *ϕ_FRQ_*, however, roughly tracks midnight in agreement with experimental results, even though the cost function used to fit our model to data had no terms involving conidiation time. In our simulations the FRQ-dependent phase of conidiation is thus dissociated from the *frq *mRNA profile which instead directly reflects the light environment, tracking dawn and dusk through its peak and trough phases.

**Figure 5:**
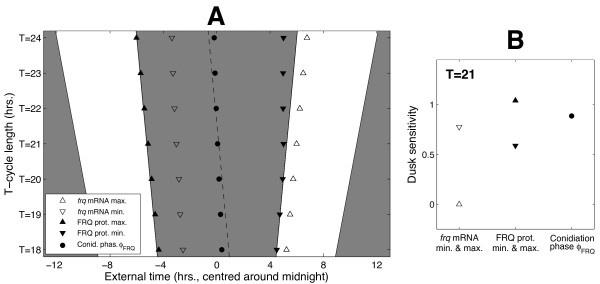
**The model reproduces the driven entrainment observed in photic T-cycles**. **A**. Simulated variation of conidiation onset with photic T-cycle length. As in Figure 4, conidiation onset was identified with the FRQ falling phase *ϕ_FRQ_*. Also plotted are the peak and trough times of *frq *mRNA and FRQ protein. White and grey regions denote light and dark respectively. The dotted line indicates a fixed period of time following dusk. **B**. Dusk sensitivities ∂*ϕ/*∂*t_DUSK _*of the phase measures shown in A for *T *= 21 (see Additional file 1, Figure S3 for the sensitivities over the full range of T-cycle lengths). ∂*ϕ_FRQ_/*∂*t_DUSK _*is close to 1: conidiation onset therefore tracks dusk.

Interestingly, our model also reproduces the driven entrainment observed experimentally in symmetric T-cycles. Figure 5A shows that for *T *in the range 18 ≤ *T *≤ 24, FRQ-dependent conidiation onset occurs roughly the same number of hours following dusk irrespective of cycle length; that is, *ϕ_FRQ_* tracks dusk. Again, like the variation of *ϕ_FRQ _*with photoperiod in 24 hr LD cycles, this is a correctly simulated system-level property that was not a direct target of the cost function. The good fits to phase data can thus be viewed as a validation of our model. For both the T-cycle and photoperiod simulations, we numerically quantified these phase variations by considering the sensitivities of *frq *mRNA and conidiation onset with respect to dawn and dusk, as described below.

#### Measuring dawn/dusk tracking using dusk sensitivity

The degree to which a circadian phase measure *ϕ* is sensitive to variations in dawn and dusk is determined by the rate of change ∂*ϕ/*∂*t_DUSK_* of *ϕ *with respect to the time *t_DUSK _*of dusk (here, *ϕ *can be conidiation onset *ϕ_FRQ_* or the times at which *frq *mRNA and FRQ protein are expressed at their minimum and maximum levels). As detailed in section 3 of Additional file 1, this sensitivity measure is bounded between 0 and 1, with a value of 1 indicating a phase that is perfectly locked to dusk and a value of 0 indicating a phase that is perfectly locked to dawn. In light-response plots such as those shown in Figures 4A and 5A, these values correspond to *ϕ *lying parallel to the lines indicating the times of dusk and dawn respectively. Intermediate values of ∂*ϕ/*∂*t_DUSK_* correspond to a systematic change in *ϕ *with photoperiod (*ϕ *non-parallel to both dusk and dawn). A sensitivity of 0.5 denotes exactly equal responses to dusk and dawn, corresponding to a clock that tracks the middle of the night.

#### Dusk sensitivities for the model

For 24 hr LD cycles, the disassociation of FRQ-dependent conidiation phase *ϕ_FRQ _*from *frq *mRNA expression is shown in terms of the corresponding dusk sensitivity measures in Figure 4B. In short and long days, the phases of peak and trough *frq *expression have dusk sensitivities close to either 0 or 1, indicating locking to dawn and dusk respectively.

Conidiation phase *ϕ_FRQ_*, however, has a sensitivity close to 0.5 in both environments, reflecting a near-zero phase change with varying photoperiod. By contrast to the systematic entrainment seen in 24 hr LD cycles, the driven behaviour of *ϕ_FRQ _*in symmetric T-cycles is quantified by a ∂*ϕ_FRQ_/*∂*t_DUSK_* value close to 1 at the intermediate value *T *= 21, indicating a dusk-driven system (see Figure 5B).

### Quantifying the effects of positive feedback

A recent computational study compared a model of the *Neurospora *clock incorporating only the central negative *frq *loop with models that also incorporated the positive *wc-1 *loop [37]. Simulations of these models - which did not explicitly consider light-signalling - suggested that the *wc-1 *loop contributes to the robustness of the system by reducing the sensitivity of the free-running period to parameter fluctuations, while allowing significant variations in oscillation amplitude [37]. Experimental work, however, suggests that decoupling the *wc-1 *loop from the *frq *loop leads to the loss of the free-running rhythm altogether [49]. Figure S1A of Additional file 1 shows that reducing positive feedback strength in our model leads to the loss of self-sustained oscillations, consistent with the experimental data.

**Figure 6:**
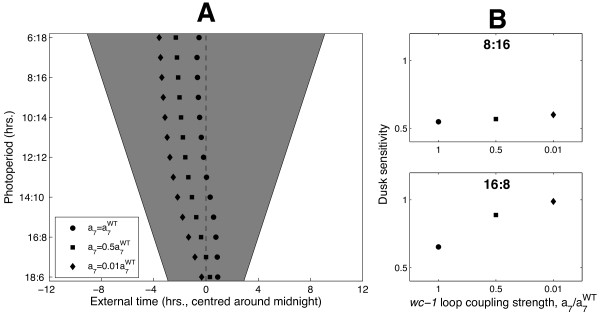
**The model predicts that the positive loop promotes systematic entrainment in long days**. **A**. The effect of varying the coupling strength of the positive feedback loop on conidiation phase *ϕ_FRQ_*. **B**. Conidiation phase dusk sensitivities ∂*ϕ_FRQ_/*∂*t_DUSK _*computed for short and long days (upper and lower plots respectively). In short days, ∂*ϕ_FRQ_/*∂*t_DUSK _*remains close to 0.5 as coupling strength is decreased, indicating systematic entrainment. In long days, however, ∂*ϕ_FRQ_/*∂*t_DUSK _*increases from 0.5 to 1 as positive feedback is abolished, quantifying the transition from an entrained to a (dusk-) driven rhythm that can be seen in A. This transition can be see in greater detail in Additional file 1, Figure S2B which shows the dependence of the sensitivity-photoperiod profile on positive feedback strength.

The good fits of our model to entrainment data (Figures 4A and 5A) did, however, suggest investigating how decoupling the *wc-1 *loop affects photoperiodicity. Figure 6A shows the variation of *ϕ_FRQ_* with photoperiod when the level of positive feedback is reduced to 50% and 1% of its wild-type value. It can be seen that decreasing the coupling strength advances phase across all photoperiods. However, the simulated decoupling mutants show qualitatively different behaviour in short days versus long days. In short days - despite a phase advance - the mutants still exhibit systematic entrainment with dusk sensitivities ∂*ϕ_FRQ_/*∂*t_DUSK _*close to the wild-type value of 0.5. In long days, by contrast, reducing the feedback strength causes a transition from systematic to dusk-driven entrainment, quantified by an increase in ∂*ϕ_FRQ_/*∂*t_DUSK_* from 0.5 to 1.

#### The wc-1 loop yields phase robustness

The phase and dusk sensitivity plots shown in Figure 6 indicate that the *wc-1 *loop may contribute to the robustness of entrained phase against photoperiod variations by enabling systematic entrainment to persist as photoperiod increases. Indeed, systematic entrainment comprises an example of robustness where a property of the whole system can be summarised using a single measure, in line with the general scheme proposed by Kitano [11]. For *Neurospora*, dawn- or dusk-locking (dusk sensitivity equal to 0 or 1) represents the least robust entrainment, which is directly driven by light, while systematic entrainment with a dusk sensitivity of 0.5 is the most robust. Following [11], if we consider variations in photoperiod *P *over a range *P*_1 _*≤ P ≤ P*_2_, then an appropriate quantitative measure of phase robustness   is(1)

where (*P*) is an evaluation function bounded between 0 and 1 measuring how the performance of the system varies with *P*. We chose an evaluation function for which (*P*_0_) = 0 denotes a clock that is locally driven (i.e. that remains dusk-or dawn-driven for small variations of *P *around *P*_0_) and (*P*_0_) = 1 denotes a clock that is locally systematically entrained (i.e. that continues to track the middle of the night under small changes to *P*). This results in a minimum phase robustness score = 0 corresponding to a clock that remains locked to either dawn or dusk as *P *varies over the entire range *P*_1_ ≤ *P *≤ *P*_2_ (global driven entrainment) and a maximum robustness score = 1 corresponding to a clock that exhibits a systematic variation of phase across the range (global systematic entrainment). The definition of robustness used here is therefore in the sense of maintaining circadian function as parameters are varied, rather than preserving the molecular dynamics of the unperturbed system [11]. The form of the evaluation function used is given in section 4 of Additional file 1.

**Figure 7:**
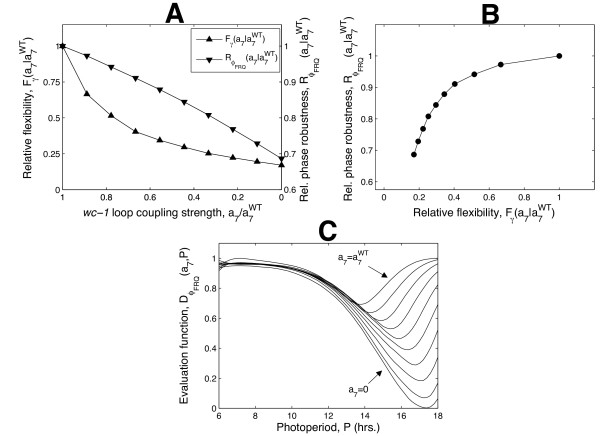
**Robustness from flexibility in the Neurospora clock**. **A**. Variations in the relative flexibility and robustness of FRQ-dependent conidiation phase with *wc-1 *loop coupling strength *a*_7_. Both measures decreases monotonically as the *wc-1 *loop is progressively decoupled from the negative *frq *loop. **B**. Relative flexibility and phase robustness are positively correlated, suggesting that one of the benefits of the increased flexibility conferred by the *wc-1 *loop is greater phase robustness against photoperiod changes. **C**. Dependence on *a*_7_ of the evaluation function  used to compute the relative robustness measure  plotted in A.  measures the extent to which entrained phase varies locally with photoperiod *P *: driven and systematic entrainment are quantified by  values of 0 and 1 respectively. In long days, decreases to 0 as *a*_7 _is reduced, causing the observed decrease in .

The robustness index defined in (1) can be used to quantify the effect of decoupling the positive *wc-1 *loop from the negative *frq *loop. The ratio of the  value for a system with modified coupling to that of the WT yields a measure of relative robustness: a score greater than 1 implies a clock that is more robust than the WT; a score less than 1 a less robust network (see Additional file 1, section 4 for details). Figure 7A shows that decoupling the *wc-1 *loop reduces the relative robustness of the modified system, as quantified by a significant decrease in the measure from 1. The corresponding changes to the evaluation function are plotted in Figure 7C. It can be seen that while the function remains close to 1 in short days, its value in long days decreases to 0 as positive feedback is removed, reflecting the transition from systematic to dusk-driven entrainment plotted in Figure 6. In this case, therefore, the overall reduction in robustness is largely attributable to the small values of the evaluation function observed for longer photoperiods.

#### Clock flexibility is increased by the wc-1 loop

The analysis above suggests that the decrease in the robustness of FRQ-dependent entrained phase *ϕ_FRQ _*observed on decoupling the *wc-1 *loop is related to a loss of flexibility, since for the decoupled system, *ϕ_FRQ _*no longer responds to dawn changes in long days. Here we confirm this hypothesis. We demonstrate that reducing the coupling strength causes a reduction in the flexibility of the outputs of the clock, where by output is taken to mean any measure of circadian behaviour that can be computed from the limit cycle attractor of the entrained system (i.e. from the periodic mRNA and protein time series).

In the following, clock flexibility is quantified using a measure based on the formalism established by Rand et al [3,40]. This considers the linearisation of the map between variations in the parameters *k *of the model and the resulting changes to the entrained limit cycle γ. Parameter variations *δk *in this scheme are vector changes, in which several parameters can be varied simultaneously, not just one-at-a time. The corresponding limit cycle changes *δ*γ are variations in the infinite-dimensional vector obtained by concatenating the periodic time series of each clock component. *δ*γ thus represents changes to the full state-space representation of the limit cycle [3,40]. As described further in section 5 of Additional file 1, it follows that the singular values of the linearised map between *δk *and *δγ* yield a quantitative measure of the extent to which combinations of random parameter perturbations - which can be considered as representing evolutionary processes - are capable of tuning the outputs of the clock. Geometrically, this map transforms the ball of all possible bounded parameter perturbations into an ellipsoid of output variations. The left singular vectors of the map are the principal axes *u_i _*of this ellipse while the corresponding singular values *σ_i_* (ordered so that *σ_i _≥ σ_i+1_*) determine the extent of the ellipse along these axes. The right singular vectors *v_i _*are the directions in parameter space that map directly onto these axes.

Within this framework, the clock is flexible if significant changes to its outputs can be obtained with relatively modest parameter changes, as measured by the singular values *σ_i_*. In particular, the sum of the singular values provides a simple flexibility measure, with large values indicating a greater relative change in the outputs for parameter perturbations of a fixed size. It should be noted that this sum is in effect a measure of global sensitivity, in the sense that it considers combined parameter changes, rather than changes to single parameters alone, and the effect of these on the whole periodic solution, not just a single output variable [50]. It follows that the change in flexibility resulting from a change in *wc-1 *loop coupling strength can be measured using the ratio of this sum in the modified network to that in the WT (see section 5 of Additional file 1 for details). Values of this relative flexibility index greater than 1 indicate a system that is more flexible than the WT; values less than 1 a less flexible clock. The variation of relative flexibility with coupling strength is plotted in Figure 7A. The corresponding normalised singular value spectra are plotted in Additional file 1, Figure S4. Clearly, flexibility decreases significantly as positive feedback strength is reduced, suggesting that the *wc-1 *loop does indeed confer greater flexibility on the network.

It can also be seen in Additional file 1, Figure S4 that for each coupling strength simulated, the dominant singular value *σ*_1_ is larger than the remaining singular values by at least an order of magnitude. This implies that the flexibility of the limit cycle is mainly in the direction of the first principal component vector *u*_1_; that is, the width of the ellipsoid of output perturbations in the direction of the longest principal axis is significantly greater than its width along the remaining axes. The loss of flexibility observed on reducing positive feedback is therefore mainly a consequence of the output ellipsoid contracting along this axis (accompanied by a roughly proportional contraction along the others). We next determined what particular dynamical behaviour this overall lower flexibility reflected, in order to test whether the observed inflexibility of *ϕ_FRQ _*was the major change, or one of many effects.

#### The wc-1 loop primarily affects the flexibility of FRQ protein phase

For a number of weakly forced circadian models, the first principal component has been found to be approximately proportional to the time derivative of the limit cycle [3,40]. This finding implies that perturbations in the direction of the first principal component result in a uniform phase change: that is, all components of the limit cycle are shifted along the time axis by the same amount with no change in amplitude. As described in section 6 of Additional file 1, this can be seen by approximating the perturbed limit cycle as a combination of phase and relative amplitude changes. In the case where the first principal component is approximately proportional to the derivative of the cycle, this yields a zero change in relative amplitude together with a uniform change in phase.

**Figure 8:**
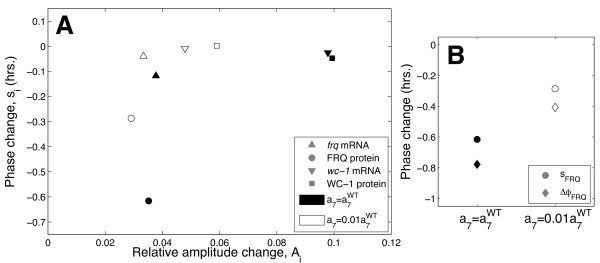
**Phase and amplitude sensitivities of the entrained system**. **A**. Phase and relative amplitude changes resulting from perturbations of the entrained clock in its maximally flexible direction. Solid and open symbols denote WT and 1% *wc-1 *loop coupling respectively. Perturbed solutions were computed for proportional parameter increases of 2%. Note that the reduction in *wc-1 *loop coupling strength causes the variation in FRQ protein phase to decrease significantly. **B**. Comparisons of the FRQ phase changes *s_FRQ _*for WT and 1% loop coupling with the corresponding changes Δ*ϕ_FRQ _*in FRQ-dependent conidiation phase. *s_FRQ _*and Δ*ϕ_FRQ _*are similar, indicating that the reduced FRQ phase sensitivity also results in reduced conidiation phase sensitivity.

Figure 8A plots the changes in phase and relative amplitude resulting from a perturbation of the WT solution along its first principal component *u*_1_, obtained through a parameter variation in the direction of the corresponding right singular vector *v*_1_. Clearly, FRQ protein undergoes a significantly greater change in phase than *frq *and *wc-1 *mRNA. Also, as can be seen in Figure 8B, the large variation in FRQ phase results in a correspondingly large shift of conidiation phase *ϕ_FRQ_*. The analysis also implies a non-uniform shift in amplitude, with *wc-1 *mRNA exhibiting a greater change compared to *frq *mRNA and FRQ protein (see Figure 8A). These phase/amplitude sensitivity calculations are confirmed by Additional file 1, Figure S5 which plots the corresponding changes to the mRNA and protein time series.

In summary, the results presented here suggest that the increased global flexibility conferred by the *wc-1 *loop is primarily an increased flexibility in conidiation phase *ϕ_FRQ_*, a particular circadian output which is a direct function of the FRQ protein dynamics. Decoupling the positive *wc-1 *loop from the central negative *frq *loop hence yields a circuit for which shifts in conidiation phase of a given size will require relatively larger perturbations to the model parameters. This implication is supported by the significant decrease in phase sensitivity observed on reducing the coupling strength plotted in Figure 8B. The phase-amplitude analysis quantifies the reduction in phase flexibility with positive feedback strength that was suggested initially by the simulations of Figure 6. Our simulations and analysis thus predict that one possible phenotypic advantage of the increased global flexibility provided by the *wc-1 *loop could be greater robustness of conidiation phase against fluctuations in photoperiod, as summarised in Figure 7B.

#### Probing the molecular mechanisms underlying phase robustness

The greater robustness conferred by the *wc-1 *loop can be understood at the level of molecular dynamics by considering the differential equation describing the dynamics of net FRQ protein, *F_T _*. As demonstrated in section 1 of Additional file 1, the rate of FRQ synthesis is given to a good approximation by the expression below:(2)

Here, *M_F _*(*t*) and *P_F_*(*t*) are the concentrations of *frq *mRNA and active FRQ, *a*_3_ is FRQ translation rate, *d*_2_ is the maximum rate of FRQ degradation and *b*_6_ is the *P_F_* concentration for which degradation occurs at 50% of its maximum rate. Under LD cycles, *frq *mRNA *M_F_* (*t*) is maintained at low levels during the night, with the exception of the rapid variations that occur around dusk and dawn (see Figure 3). It follows from the form of (2) that if the FRQ degradation rate is strongly saturated (*P_F _*is large compared to *b*_6_), the overall rate of loss of FRQ will be roughly constant during the night. FRQ protein level will hence decrease linearly with time over this period. Furthermore, a constant rate of FRQ loss means that the LD cycle can only affect FRQ levels through the acute light responses of *M_F_* (*t*). Consequently, the rapid induction of *frq* transcription just after dawn will cause the FRQ synthesis rate *Ḟ_T_* to increase through 0, resulting in a *F_T_* minimum near dawn. Conversely, the rapid decrease in *M_F_* (*t*) just after dusk will cause *Ḟ_T_* to decrease through 0, producing a *F_T_* maximum near dusk. The combined effects yield a FRQ profile that decays approximately linearly from a peak near dusk to a trough near dawn.

A simple measure of the extent to which FRQ degradation is saturated is provided by the corresponding saturation index , with values close to 1 indicating strong saturation [51]. The average value *D_SI _*of this index during the night then provides a measure of how close the FRQ loss rate is to a constant, with the maximum value of 1 denoting complete saturation between dusk and dawn. It can be seen in Additional file 1, Figure S6 that for all photoperiods, the WT has relatively high *D_SI _*values indicating strong saturation of FRQ degradation in the dark. This result is consistent with the FRQ profiles plotted previously in Figure 3 for which FRQ decreases roughly linearly from a dusk-tracking peak to a dawn-tracking trough, resulting in FRQ-dependent conidiation phase *ϕ_FRQ _*coinciding with the middle of the night (cf. Figure 4). Figure S6 also shows that reducing the coupling of FRQ to the *wc-1 *loop causes significant decreases in *D_SI _*across all photoperiods. The resulting nonlinear dark FRQ profiles move the position of conidiation phase nonuniformly across photoperiods in comparison to the WT, with an enhanced sensitivity to dusk as photoperiod is increased resulting in the lower phase robustness of the decoupling mutants.

The *wc-1 *loop thus provides a mechanism for tuning the saturation level of FRQ degradation so as to obtain near-linear dark FRQ profiles for which the peaks and troughs move together with dusk and dawn. This in turn yields a flexible FRQ-dependent conidiation phase *ϕ_FRQ _*that responds to both dusk and dawn signals and can therefore track the middle of the night across photoperiods.

## Discussion

### A model of the Neurospora clock simulating photoentrainment of the FRQ-WC oscillator

Together with *Drosophila melanogaster *and *Arabidopsis thaliana*, *Neurospora crassa *has become a key organism in the computational modelling and analysis of circadian networks. For example, models based on the central negative *frq *loop have been used to investigate the biochemical mechanisms underlying the temperature compensation of the clock [38], together with the effects of molecular noise on the robustness of free-running and entrained rhythms [35]. More complex models incorporating the positive *wc-1 *loop have enabled hypotheses to be made regarding the means by which FRQ upregulates WC-1 [37] and inhibits *frq *transcription [39], as well as the possible functional advantages conferred by the positive loop [37] and the parallel pathways comprising the negative loop [8]. These models have proved useful tools in uncovering the design principles of the clock, while also generating a number of important experimental predictions.

Within this framework, we have presented here a mathematical model for the circadian clock of *Neurospora crassa *based on the central FRQ-WC oscillator, incorporating both the *frq *and *wc-1 *loops. While previous models of the *Neurospora *clock have modelled the effect of light through direct changes to transcription or degradation rates, we incorporated elements of the light-signalling pathway explicitly in order to be able to quantitatively examine the relationship between network structure and entrained phase. This greater level of biochemical detail enabled us to obtain good fits to experimental data in both free-running and entrained conditions. In particular, we were able to simulate the disassociation between light-driven *frq *mRNA and photoperiodic FRQ protein reported experimentally [16]. While *frq *trough and peak phases are both light-driven in our simulations, FRQ-dependent conidiation phase tracks the middle of the night. The model also reproduces the dusk-driven behaviour observed in symmetric photic T-cycles [15]. In addition, we introduced a novel measure assessing the relative sensitivity of phase to changes in the times of dawn and dusk which provided a quantitative means for distinguishing between the systematic and driven conidiation observed in the photoperiod and T-cycle simulations respectively.

### An example of robustness from flexibility

Theoretical and experimental studies have suggested that one of the benefits conferred by multiple feedback loops is increased evolutionary flexibility, with the number of key functionalities of the system that can be tuned independently of one another increasing with the number of loops [3,7,8,40,52]. This greater flexibility can in turn lead to greater robustness of the system against environmental and genetic perturbations [4,6,8,10]. In a previous paper, we gave an example of robustness following from flexibility for the *Neurospora *system using a temperature-dependent variant of the model presented here. In that study, we proposed that the presence of two parallel negative feedback loops with opposing temperature dependence controlling the production of the FRQ isoforms enables low dimensional tuning of the entrainment phase-temperature relationship, facilitating buffering of the clock against seasonal temperature fluctuations [8].

Here, we were interested in investigating how the positive *wc-1 *loop affects the clock. Previous computational studies have examined the role of positive feedback on the control of free-running period and amplitude [36,37]. In our model, however, decoupling the *wc-1 *loop results in arrhythmicity, in line with experimental observations [49]. More generally, entrained phase rather than free-running period *per se *is expected to have selective value in the natural environment [53,54], thereby identifying phase as a key systems-level output for computational studies [8,55,56]. This led us to examine the effect of decoupling the loop on the robustness of conidiation phase to photoperiod variations. We introduced a robustness index based on the framework proposed by Kitano [11], with maximum robustness being attributed to a system that exhibits systematic entrainment over the full range of photoperiods considered, and minimum robustness to a system that is dusk- or dawn-driven over the same range. Using this measure, we found that removing positive feedback leads to a decrease in phase robustness and that this is a consequence of a transition from systematic to dusk-driven entrainment in long days.

For the next part of the analysis, we introduced a simple scalar measure quantifying the variations in the outputs of the clock resulting from parameter perturbations mimicking evolutionary processes. This enabled us to demonstrate that the *wc-1 *loop enhances the evolutionary flexibility of the clock, consistent with the predictions of previous theoretical studies [3,40]. Using a novel method that is applicable to any entrained biological oscillator, we then computed phase and amplitude sensitivities for perturbations of the clock in its most flexible direction. These sensitivities can be viewed as entrained versions of the period-amplitude sensitivities commonly used to assess the robustness of free-running clocks [36,37,46]. However while the latter are usually computed from scalar perturbations (variations of individual parameters) simulating single mutations, the phase-amplitude sensitivities were obtained by considering vector perturbations that simulate the parameter changes most likely to affect system behaviour under evolutionary changes [3,4,40]. The calculated sensitivities showed that the greater flexibility provided by the *wc-1 *loop is predominately manifested as greater flexibility of conidiation phase, a key FRQ-dependent circadian output. Finally, we quantified the molecular basis of the enhanced flexibility - and the resulting robustness of the clock's photoentrainment - demonstrating that the *wc-1 *loop provides a low-dimensional mechanism for optimally tuning the extent to which FRQ protein degradation is saturated.

To summarise, our results imply that one of the possible benefits of the increased flexibility conferred by the *wc-1 *loop is the persistence of systematic entrainment in long days, contributing to the robustness of the clock with respect to long-term changes in photoperiod. Taken together with previous work, this result could be interpreted as an additional specific example of how increased loop complexity can confer greater flexibility on a cellular circuit, in turn promoting robustness against environmental fluctuations [10].

### Predictions and further model development

The work presented here, and previously in [8], predict important roles for the *wc-1 *and parallel *frq *loops in maintaining circadian function. This does not necessarily imply that all observed feedback loops in the *Neurospora *circuit need be critical for adaptive clock behaviour. It does, however, demonstrate that detailed models can provide testable predictions regarding the relationship between the constituent loops of the clock and core circadian outputs. The suggestion that decoupling the positive *wc-1 *loop from the central feedback loop will abolish systematic entrainment in long days comprises such a prediction and would provide a good test of our model. This prediction could be tested directly by assessing conidiation rhythms in the mutant *frq *strain *frq*-S885/7N, for which WC-1 expression is significantly reduced as a consequence of reduced FRQ phosphorylation [49].

In addition, our model also predicts that the loss of free-running rhythmicity observed in the *frq*-S885/7N strain arises as a consequence of a supercritical Hopf bifurcation (Additional file 1, Figure S1A). The supercritical Hopf bifurcation is one of three typical mechanisms by which periodic oscillations can be destroyed as a system parameter - in this case coupling strength - is altered. For the supercritical Hopf bifurcation of Figure S1A, decreasing the parameter past a certain critical value collapses the DD limit cycle onto an equilibrium point, with the amplitude of oscillations decreasing continuously to zero as this happens. The alternative mechanisms are: i) the destruction of the limit cycle through its collision with an unstable limit cycle generated by a subcritical Hopf bifurcation; and ii) the SNIC (saddle node on invariant circle) bifurcation in which stable and unstable equilibrium points are created simultaneously on the limit cycle [57]. In contrast to the supercritical Hopf, both the subcritical Hopf and SNIC are characterised by a sudden loss of rhythmicity without significant amplitude changes at the bifurcation point [57]. In addition, the SNIC has a distinct experimental signature in which the rhythm freezes at a well-defined phase as the bifurcation is approached.

The particular mechanism that causes arrhythmicity in *frq*-S885/7N could therefore - in principle be ascertained using the strain *frq*-S885/7N;*qa-wc-1 *which exhibits QA-induced rhythmic oscillations as a consequence of increased WC-1 expression [49]. Such an experiment would involve observing how the oscillation varied over a range of lower QA concentrations. A progressive fall in rhythmic amplitude as QA concentration is reduced - accompanied by modest changes to both period and phase (cf. Additional file 1, Figure S1B) - would suggest the supercritical Hopf bifurcation predicted by our model. By contrast, a sudden loss of rhythmicity over a narrow range of QA concentrations without a significant amplitude change would be incompatible with our model in its current form. This would instead indicate that either the subcritical Hopf or SNIC bifurcation was responsible for the destruction of the DD limit cycle, with a freezing of phase near the bifurcation point distinguishing the SNIC. In this case, the particular mechanism identified could be used as a target for further model development.

Finally, although our results show that much of the behaviour of the clock in periodic photic cycles can be accurately modelled with the core FRQ-WC oscillator, we note that our model is unable to reproduce some of the responses to shorter light intervals. Specifically, it cannot stably entrain to LD cycles with photoperiods less than 6 hrs, and is also unable to reproduce the particular form of the type 0 phase response curve (PRC) that has been observed for strong resetting cues in some experiments [41,58]. The expected effect of the VVD protein in modulating the circadian gating of light responses may in part account for these discrepancies. This suggests the inclusion of *vvd *as a suitable next iteration of the model, with PRCs and entrainment to very short days corresponding targets for model validation.

## Conclusions

The multi-loop structure of the *Neurospora *clock provides a paradigm example of the extent to which circadian clocks can diverge from the simple delayed negative feedback loop that will reliably oscillate. As experiments lead to the discovery of further clock components and the connections between them in *Neurospora *and other key organisms, mathematical modelling and analysis techniques will become increasingly useful tools in the quantitative analysis of circadian networks. Part of this program will involve the development of ever more detailed models of the complex topologies characteristic of these systems, together with the development of robust algorithms to fit the models to experimental data.

Furthermore, the continuing use of clocks to elucidate the design principles of cellular circuits will require the development of biologically realistic indices of core system-level properties - such as the flexibility and robustness measures presented here -together with analytical tools for their implementation. As an example of this, we anticipate that the global phase-amplitude sensitivity analysis method introduced in this work could prove a useful tool for identifying the particular components of a complex clock network most likely to exhibit functional changes.

## Methods

### Modelling and parameter fitting

The model equations are given in section 1 of Additional file 1 together with descriptions of their derivation. As in previous clock models, Michaelis-Menten kinetics were used to describe enzyme-mediated degradation of mRNA and active protein while Hill functions were used to model transcriptional activation and inhibition. These equations are taken to abstract sets of more elementary reactions whose biochemical details are unknown [5,7,28,33-36,38,39,46,59-62].

A relative novelty of the model is the way in which the protein pathways have been represented. Many computational models of circadian networks employ sequences of protein modifications (e.g. phosphorylation or nuclear transport) to generate the delays necessary for autonomous oscillations to be produced [61]. We used an alternative, generalised method of representing these delays. This considers the rate at which a protein is converted into its active form to be a weighted sum of the corresponding mRNA levels over the preceding time interval [34,63]. The weights in this sum are the distribution of times for the protein to be modified into its active form; a discrete distribution - concentrated at just one value - corresponds to a single, fixed delay between the translation of a protein and its effect on a downstream gene [64]. Here, we used a continuous distribution (the gamma function) capable of mimicking a variety of biologically plausible delays [65]. A significant advantage of this approach is a marked reduction in complexity as each of the individual parameters representing conversion and degradation of intermediate protein species are replaced by two global parameters governing the form of the gamma function [8,63,65]. For the model considered here, the total number of kinetic parameters was reduced to 33 from a potential maximum value of 45. In addition, the use of a gamma-distributed delay has the advantage of greatly simplifying the analysis of the corresponding set of equations compared to a discrete delay [63,65].

In all, the model comprises five coupled deterministic integrodifferential equations with a total of 36 parameters (the 33 unknown kinetic parameters together with 3 fixed parameters specifying the light input). The large number of unknown model parameters represented a significant challenge in terms of data-fitting, particularly as the free-running and entrained clock have qualitatively different dynamics (while quasi-sinusoidal oscillations are observed in DD, *frq *and *wc-1 *mRNA exhibit slow-fast dynamics closer to that of a relaxation oscillator in LD due to acute light responses in these genes). As a consequence of this dual dynamic behaviour - coupled with the significant variability of experimental time courses - we employed a bipartite optimisation method based on minimising a qualitative cost function, rather than attempting to fit directly to data [46].

The cost function we used assessed the goodness-of-fit of the model to both DD and LD experimental time series, based on reproducible circadian measures such as free-running period and the times at which mRNA and protein levels reach their minimum and maximum values [7,8,46,47]. Low cost scores correspond to parameter sets that give a good qualitative match to these target features. The parameter set yielding the smallest cost score was used to generate the simulations of the wild-type clock used in this study. A detailed account of the optimisation technique employed - including a full description of the cost function - is given in section 2 of Additional file 1. The values of the optimal parameter set are listed in Table 1. Simulations of *wc-1 *loop uncoupling were obtained by reducing the parameter *a*_7 _controlling the upregulation of WC-1 production by FRQ from its wild-type value *a*_7_*^WT ^*(see Additional file 1, equation (S.4)).

**Table 1:** Optimal model parameters

Parameter, *k_j_*	Description	Value
*a*_1 _(h^-1^)	Max. rate: WC-1* upregulated *frq *transcription	8.3450
*a*_2 _(h^-1^)	Max. rate: WC-1 upregulated *frq *transcription	3.7925
*a*_3 _(h^-1^)	FRQ translation rate	0.3154
*a*_4 _(h^-1^)	Basal *wc-1 *transcription rate	0.6787
*a*_5 _(h^-1^)	Max. rate: WC-1* upregulated *wc-1 *transcription	10.0718
*a*_6 _(h^-1^)	Basal WC-1 translation rate	6.6644
*a*_7 _(nM^-1^h^-1^)	FRQ upregulated WC-1 translation rate	2.4695
*b*_1 _(nM^-1^)	Michaelis constant: repression of WC-1* upregulated *frq *transcription	4.1472
*b*_2 _(nM)	Michaelis constant: WC-1* upregulated *frq *transcription	0.1560
*b*_3 _(nM^-1^)	Michaelis constant: repression of WC-1 upregulated *frq *transcription	0.7149
*b*_4 _(nM^)^	Michaelis constant: WC-1 upregulated *frq *transcription	2.9415
*b*_5 _(nM)	Michaelis constant: *frq *mRNA degradation	4.1075
*b*_6 _(nM)	Michaelis constant: degradation of active FRQ	0.4715
*b*_7 _(nM)	Michaelis constant: WC-1* upregulated *wc-1 *transcription	3.5676
*b*_8 _(nM)	Michaelis constant: *wc-1 *mRNA degradation	0.5805
*b*_9 _(nM)	Michaelis constant: degradation of active WC-1	7.0233
*b*_10 _(nM)	Michaelis constant: degradation of WC-1*	0.8218
*d*_1 _(h^-1^)	Max. rate: *frq *mRNA degradation	7.4608
*d*_2 _(h^-1^)	Max. rate: degradation of active FRQ	0.4405
*d*_3_ (h^-1^)	Max. rate: *wc-1 *mRNA degradation	2.1710
*d*_4 _(h^-1^)	Max. rate: degradation of active WC-1	3.0883
*d*_5 _(h^-1^)	Max. rate: degradation of WC-1*	23.3120
*f_1 _*(h^-1^)	Delay parameter: FRQ → active FRQ conversion	0.1962
*f_2 _*(h^-1^)	Delay parameter: WC-1 → active WC-1 conversion	0.1317
γ_1 _(h^-1^)	Loss rate: FRQ → active FRQ conversion (deg. rate of intermediates)	0.0422
γ_2 _(h^-1^)	Loss rate: WC-1 → active WC-1 conversion (deg. rate of intermediates)	0.0244
*r*_1 _(h^-1^)	Rate of active WC-1 → WC-1* conversion	5.1759
*r*_2 _(h^-1^)	Rate of WC-1* → active WC-1 conversion	5.0326
*n*	Hill coefficient: WC-1* upregulated *frq *transcription	1.0168
*m*	Hill coefficient: WC-1 upregulated *frq *transcription	2.8134
*k*	Hill coefficient: WC-1* upregulated *wc-1 *mRNA transcription	1.4135
*g*	Hill coefficient: repression of WC-1* upregulated *frq *transcription	1.2730
*h*	Hill coefficient: repression of WC-1 upregulated *frq *transcription	3.6978

Parameter values yielding the optimal value of the cost function used to fit the model to experimental data. WC-1* denotes light-induced active WC-1.

### Simulations and software

Solutions of the model were obtained by converting integrodifferential equations into equivalent sets of ordinary differential equations, allowing them to be integrated using standard solvers (see Additional file 1, section 1 for details). Conidiation phase *ϕ_FRQ _*was computed as the unique solution of:

Here, *F_T _*(*t*) is the FRQ protein profile,  and  represent the peak and trough values of FRQ protein and *α* is a tuning parameter such that *ϕ_FRQ_* coincides with midnight in 12:12 LD cycles. For all simulations presented in this work, *α* was fixed at the value 0.15.

Model simulations and sensitivity analyses were carried out with custom software developed in MATLAB (Mathworks, Cambridge, UK). Parameter optimisation was implemented by converting numerical routines initially written in MATLAB into C++ and running the code on a task farm computer consisting of 17 × 2.0 GHz 2-way IBM Opteron nodes. All routines used are available by request.

## Authors' contributions

The model was constructed by OEA, AJM and DAR. Parameter optimisation was carried out by OEA and PEB. Dusk sensitivities and robustness measures were developed by OEA. Flexibility measurements and phase-amplitude analyses were adapted by OEA from analytical techniques proposed by DAR. All simulations and model analyses were carried out by OEA. The paper was written by OEA and AJM. AJM provided biological details, advice and detailed criticism. All authors read and approved the final manuscript.

## Acknowledgements

The authors would like to thank Sanyi Tang and James Locke for fruitful discussions on clock modelling and parameter optimisation, Isabelle Carré for biological criticisms and John O'Neill and Laura Dixon for useful comments on the manuscript. The Centre for Systems Biology at Edinburgh is a Centre for Integrative Systems Biology (CISB) funded by BBSRC and EPSRC, reference BB/D019621/1. Funding at Warwick was provided by the BBSRC, EPSRC and EU (BioSim Network Contract No. 005137). This work has made use of the resources provided by the Edinburgh Compute and Data Facility (ECDF) http://www.ecdf.ed.ac.uk/. The ECDF is partially supported by the eDIKT initiative http://www.edikt.org.uk/edikt2/. Additional high-computing facilities were provided by the Centre for Scientific Computing at the University of Warwick http://www.csc.warwick.ac.uk/.

## Supplementary Material

Additional file 1 **Supplementary Information**. This file contains Supplementary Figures S1-S6 together with details of the modelling, parameter optimisation and sensitivity analysis methods used in this work. Click here for file
