# Supplementary material for: Robustness from flexibility in the fungal circadian clock
Source: BMC Syst Biol. 2010 Jun 24;4:88. doi: 10.1186/1752-0509-4-88 (PMC2913929; doi:10.1186/1752-0509-4-88)
Supplement: Additional file 1 — Supplementary Information. This file contains Supplementary Figures S1-S6 together with details of the modelling, parameter optimisation and sensitivity analysis methods used in this work. [file 1752-0509-4-88-S1.PDF]

Supplementary Information for ***Robustness from flexibility in the fungal circadian clock.***, O.E. Akman et al.

Contents

1 Mathematical modelling **2**

1.1 Model equations . . . 2

1.2 Model simulations . . . 3

2 Parameter optimisation **4**

2.1 Specification of the cost function . . . 4

2.2 Description of the optimisation algorithm . . . 6

2.3 Comparison of experimental and *in silico* protein degradation rates . . . 7

3 Quantifying the sensitivity of phase to changes in dawn and dusk **8**

4 Measuring the robustness of phase to changes in photoperiod **9**

5 Quantifying network flexibility **10**

5.1 Measuring the flexibility of circadian outputs . . . 10

5.2 Calculating the relative flexibility of the clock network . . . 11

6 Phase and amplitude variations for the entrained limit cycle **12**

References **14**

Supplementary Figures **16**

1 Mathematical modelling

1.1 Model equations

The differential equations used to generate the simulations presented in this study are the following:

$$\begin{aligned}
\dot{M}_F &= a_1 \frac{\left(P_W^L\right)^n}{\left(1+\left(\frac{P_F}{b_1}\right)^g\right)\left(\left(P_W^L\right)^n + b_2^n\right)} + a_2 \frac{\left(P_W\right)^m}{\left(1+\left(\frac{P_F}{b_3}\right)^h\right)\left(\left(P_W\right)^m + b_4^m\right)} \\
&\quad - d_1 \frac{M_F}{M_F + b_5} && \text{(S.1)} \\
\dot{P}_F &= a_3 \int_{-\infty}^{t} M_F(s)\, g_{f_1}(t-s)\, e^{-\gamma_1 (t-s)} ds - d_2 \frac{P_F}{P_F + b_6} && \text{(S.2)} \\
\dot{M}_W &= a_4 + a_5 \frac{\left(P_W^L\right)^k}{\left(P_W^L\right)^k + b_7^k} - d_3 \frac{M_W}{M_W + b_8} && \text{(S.3)} \\
\dot{P}_W &= \int_{-\infty}^{t} M_W(s)\left(a_6 + a_7 P_F(s)\right) g_{f_2}(t-s)\, e^{-\gamma_2 (t-s)} ds - d_4 \frac{P_W}{P_W + b_9} \\
&\quad - r_1 \theta(t) P_W + r_2 P_W^L && \text{(S.4)} \\
\dot{P}_W^L &= r_1 \theta(t) P_W - r_2 P_W^L - d_5 \frac{P_W^L}{P_W^L + b_{10}}. && \text{(S.5)}
\end{aligned}$$

Here, the variables M_F and M_W denote the concentrations of *frq* and *wc-1* mRNA respectively. P_W is the concentration of active WC-1 and P_W^L represents the concentration of light-induced active WC-1, termed WC-1*. The variable P_F denotes the level of active FRQ. These concentrations are taken to reflect the number of moles per cell and are reported in arbitrary units (a.u.), due to the fact they are not fitted directly to experimental expression levels [1–4].

The repressive action of FRQ on *frq* transcription occurs through the formation of FRQ:WCC complexes followed by FRQ-mediated clearance of the WCC from the nucleus [4]. This process is therefore modelled using terms based on noncompetitive inhibition (equation (S.1)) [2]. The model also includes the positive feedback loop in which FRQ enhances the accumulation of WC-1 (equation (S.4)). Light acts on the system through a smooth function $\theta(t)$ modelling a T-periodic light-dark cycle which switches rapidly between 0 and a maximum value θ_{amp} equal to 1 at dawn ($t = t_{DAWN}$), and from θ_{amp} back to 0 at dusk ($t = t_{DUSK}$):

$$\theta(t) = \frac{\theta_{amp}}{4}\left(1 + \tanh\left(T\left(\text{mod}(t,T) - t_{DAWN}\right)\right)\right)\left(1 - \tanh\left(T\left(\text{mod}(t,T) - t_{DUSK}\right)\right)\right).$$

This term acts in two ways. Firstly, it raises the forward rate of the reaction $P_W \rightleftharpoons P_W^L$ in equations (S.4) and (S.5): this models the rise in the relative concentration of FAD-bound WC-1 in complex with the LREs at the *frq* promoter observed with increasing light levels [5], and the resulting enhanced transcription of *frq* [6, 7]. Through this mechanism, it also increases the transcription rate of *wc-1* through the second term of (S.3), reflecting the loss of *wc-1* light-responses in *wc-1* mutant backgrounds [8].

In equations (S.2) and (S.4), the distribution of times required for FRQ and WC-1 protein to be translated and converted into their active forms (through, for example, phosphorylation or nuclear transport) is assumed to be a gamma function with integer scale parameter f_i:

$$g_{f_i}(t) = f_i^2 t e^{-f_i t}.$$

This distribution, commonly referred to as the Erlang distribution, has mean delay $2/f_i$ - representing the average conversion time - and variance $2/f_i^2$ - representing the mean deviation from the average. The term $e^{-\gamma_i t}$ that post-multiplies the Erlang term in (S.2) and (S.4) corresponds to the loss of protein during this process, with γ_i quantifying the rate of loss [9].

1.2 Model simulations

The integrodifferential equations (S.1)-(S.5) can be converted into a set of ordinary differential equations (ODEs) using the linear chain trick [9]. Introducing the auxiliary variables $\left\{E_1^F, E_2^F\right\}$ and $\left\{E_1^W, E_2^W\right\}$ defined by

$$\begin{aligned} E_j^F(t) &= \frac{a_3}{f_1}\int_{-\infty}^{t} G_{f_1}^{j-1}(t-s)\, e^{-\gamma_1(t-s)} M_F(s)\, ds \\ E_j^W(t) &= \frac{1}{f_2}\int_{-\infty}^{t} G_{f_2}^{j-1}(t-s)\, e^{-\gamma_2(t-s)} \left(a_6 + a_7 P_F(s)\right) M_W(s)\, ds \end{aligned}$$

where

$$G_{f_i}^m(t) = \frac{t^m}{m!} f_i^{m+1} e^{-f_i t}$$

enables the equations to be expressed in the form below:

$$\begin{aligned} \dot{M}_F &= a_1 \frac{\left(P_W^L\right)^n}{\left(1+\left(\frac{P_F}{b_1}\right)^g\right)\left(\left(P_W^L\right)^n + b_2^n\right)} + a_2 \frac{\left(P_W\right)^m}{\left(1+\left(\frac{P_F}{b_3}\right)^h\right)\left(\left(P_W\right)^m + b_4^m\right)} \\ &\quad - d_1 \frac{M_F}{M_F + b_5} \qquad \text{(S.6)} \\ \dot{E}_1^F &= a_3 M_F - \left(f_1 + \gamma_1\right) E_1^F \qquad \text{(S.7)} \\ \dot{E}_2^F &= f_1 E_1^F - \left(f_1 + \gamma_1\right) E_2^F \qquad \text{(S.8)} \\ \dot{P}_F &= f_1 E_2^F - d_2 \frac{P_F}{P_F + b_6} \qquad \text{(S.9)} \\ \dot{M}_W &= a_4 + a_5 \frac{\left(P_W^L\right)^k}{\left(P_W^L\right)^k + b_7^k} - d_3 \frac{M_W}{M_W + b_8} \qquad \text{(S.10)} \\ \dot{E}_1^W &= \left(a_6 + a_7 P_F\right) M_W - \left(f_2 + \gamma_2\right) E_1^W \qquad \text{(S.11)} \\ \dot{E}_2^W &= f_2 E_1^W - \left(f_2 + \gamma_2\right) E_2^W \qquad \text{(S.12)} \\ \dot{P}_W &= f_2 E_2^W - d_4 \frac{P_W}{P_W + b_9} - r_1 \theta(t) P_W + r_2 P_W^L \qquad \text{(S.13)} \\ \dot{P}_W^L &= r_1 \theta(t) P_W - r_2 P_W^L - d_5 \frac{P_W^L}{P_W^L + b_{10}}. \qquad \text{(S.14)} \end{aligned}$$

In this formulation, the delays between the translation of FRQ and WC-1 protein and their conversion into active forms - as described by the integrodifferential equations (S.2) and (S.4) - are generated by chains of linear ODEs involving the auxiliary variables. The latter can therefore be thought of as intermediate protein species (e.g. phosphorylated or nuclear/cytoplasmic forms), with the total amounts of FRQ and WC-1 protein given by $F_T = \sum_{i=1}^{2} E_i^F + P_F$ and $W_T = \sum_{i=1}^{2} E_i^W + P_W + P_W^L$ respectively [2]. In particular, summing equations (S.7)-(S.9) yields the following equation for total FRQ synthesis:

$$\dot{F}_T = a_3 M^F - \gamma_1 \left(E_1^F + E_2^F\right) - d_2 \frac{P_F}{P_F + b_6}. \qquad \text{(S.15)}$$

Table 1 of the main paper shows that $\gamma_1 \ll 1$. Consequently, (S.15) is well approximated by equation (2) of the main paper.

2 Parameter optimisation

Parameter values $k = (k_1, \ldots, k_s)$ of the model giving a qualitative match to experimental data were obtained by minimising a cost function $C(k)$ comprising a sum of terms measuring the agreement between the model and certain key reproducible features of the data. Following [10], the weighting of each term in the cost function was chosen to yield an O(1) contribution for an experimentally acceptable error. The terms in the cost function were evaluated by numerically solving equations (S.6)-(S.14) over 300 hrs in a simulated 12:12 light dark (LD) cycle, followed by 300 hrs in simulated constant darkness (DD). In each 300 hr interval, the first 204 hrs were discarded as transients.

2.1 Specification of the cost function

The cost function comprises a sum of nine individual terms, each of which corresponds to a set of target qualitative features:

$$\begin{aligned} C &= C_{\tau_{LD}} + C_{OSC_{LD}} + C_{\phi_{LD}} + C_{ALR_{LD}} + C_{PF_{LD}} + C_{\tau_{DD}} \\ &\qquad + C_{OSC_{DD}} + C_{\phi_{DD}} + C_{PF_{DD}}. \end{aligned} \tag{S.16}$$

Here, we provide a description of each of these terms. Throughout this section, simulated time series of *frq* mRNA, *wc-1* mRNA, FRQ protein and WC-1 protein will be denoted by the variables $y_f(t)$, $y_w(t)$, $y_F(t)$ and $y_W(t)$ respectively. The phases at which these variables reach their peak and trough values will be written as θ_i^P and θ_i^T, with $A_i = y_i\left(\theta_i^P\right) - y_i\left(\theta_i^T\right)$ the corresponding oscillation amplitudes and Δ_i^P the times between successive peaks. The operators $\langle . \rangle_{LD}$ and $\langle . \rangle_{DD}$ calculate the average of their arguments over the last 96 hrs of simulation in LD and DD, while $\sigma(.)_{LD}$ and $\sigma(.)_{DD}$ compute the corresponding standard deviations. $[.]_{DD}^B$ and $[.]_{DD}^E$ return the first and last instances of their arguments over the last 96 hours of simulated DD (so, for example, $\left[\theta_F^T\right]_{DD}^B$ calculates the phase of the first FRQ trough encountered over this interval). Finally, L_0 and L_1 denote the times of dusk and dawn.

The first term $C_{\tau_{LD}}$ in (S.16) measures the difference between the period of the solution generated in LD and the target entrained period of 24 hrs:

$$C_{\tau_{LD}} = \sum_{i=f,w,F} \left\langle \left(\left(24 - \Delta_i^P\right)/0.25\right)^2 \right\rangle_{LD}.$$

The second term $C_{OSC_{LD}}$ penalises large variations in successive peaks, corresponding to solutions that are not properly entrained. It also penalises solutions with very small (O(10^{-1})) oscillation magnitudes, as these are unable to generate the large changes in expression level resulting from the system's acute light-responses:

$$C_{OSC_{LD}} = \sum_{i=f,w,F} \left[\left(\sigma\left(y_i\left(\theta_i^P\right)\right)_{LD} / 0.025\langle y_i\left(\theta_i^P\right)\rangle_{LD}\right)^2 + \left(1.5/\langle A_i\rangle_{LD}\right)^2 \right].$$

The third term $C_{\phi_{LD}}$ calculates the difference between simulated and target phases in LD. It measures the errors for the peak phases of *frq* and *wc-1* mRNA (target phases: 0.5 hrs after dawn)

together with the trough phase of *frq* mRNA (target phase: 3 hrs after dusk) and the peak and trough phases of FRQ protein (target phases: 2 hrs after dusk and dawn respectively). $C_{\phi_{LD}}$ has the form

$$
\begin{aligned}
C_{\phi_{LD}} &= F_{\phi_{LD}}\left(\theta_f^P, L_1+0.5, 0.5\right) + F_{\phi_{LD}}\left(\theta_f^T, L_0+3, 1\right) + F_{\phi_{LD}}\left(\theta_F^T, L_1+2, 0.5\right) \\
&\quad + F_{\phi_{LD}}\left(\theta_F^P, L_0+2, 0.5\right) + F_{\phi_{LD}}\left(\theta_w^P, L_1+0.5, 0.5\right),
\end{aligned}
$$

where:

$$
F_{\phi_{LD}}\left(\theta_1, \theta_2, E_\theta\right) = \left\langle \left(\left(\theta_1-\theta_2\right)/E_\theta\right)^2 \right\rangle_{LD} + \sigma_{LD}\left(2\left(\theta_1-\theta_2\right)/E_\theta\right)^2.
$$

The fourth term $C_{ALR_{LD}}$ checks that *frq* mRNA exhibits both acute dawn and dusk responses and that *wc-1* mRNA exhibits an acute dawn response, as reported in [7]:

$$
\begin{aligned}
C_{ALR_{LD}} &= \left\langle \left(\frac{\frac{2}{3}A_f}{y_f\left(\theta_f^P\right) - y_f\left(\theta_f^P - 2\right)} \right)^2 \right\rangle_{LD} + \left\langle \left(\frac{\frac{1}{2}A_f}{y_f\left(\theta_f^P\right) - y_f\left(\theta_f^P + 2\right)} \right)^2 \right\rangle_{LD} \\
&\quad + \left\langle \left(\frac{\exp\left(\frac{5}{3}\right)}{\exp\left(5\left(y_f\left(L_0-1\right) - y_f\left(L_1+1\right)\right)/A_F\right)} \right)^2 \right\rangle_{LD} \\
&\quad + \left\langle \left(\frac{\frac{9}{10}A_w}{y_w\left(\theta_w^P\right) - y_w\left(\theta_w^P - 2\right)} \right)^2 \right\rangle_{LD} + \left\langle \left(\frac{\frac{3}{4}A_w}{y_w\left(\theta_w^P\right) - y_w\left(\theta_w^P + 2\right)} \right)^2 \right\rangle_{LD}.
\end{aligned}
$$

The fifth term $C_{PF_{LD}}$ ensures that: 1) *frq* and *wc-1* mRNA profiles stay close to an equilibrium during the light phase of LD cycles; 2) *wc-1* mRNA returns to a near-baseline level following its acute dawn response; and 3) *frq* mRNA does not converge to an equilibrium during the dark phase. It is given by the expression:

$$
\begin{aligned}
C_{PF_{LD}} &= \left\langle \left(\frac{10\left(y_f\left(\theta_f^P+2\right) - y_f\left(L_0-1\right)\right)}{A_f} \right)^2 \right\rangle_{LD} + \left\langle \left(\frac{10\left(y_w\left(\theta_w^P+2\right) - y_w\left(L_0-1\right)\right)}{A_w} \right)^2 \right\rangle_{LD} \\
&\quad + \left\langle \left(\frac{10\left(y_w\left(\theta_w^T\right) - y_w\left(L_0\right)\right)}{A_w} \right)^2 \right\rangle_{LD} + \left\langle \left(\frac{\frac{1}{10}A_f}{y_f\left(L_1-1\right) - y_f\left(\theta_f^T\right)} \right)^2 \right\rangle_{LD}.
\end{aligned}
$$

The sixth term $C_{\tau_{DD}}$ measures the deviation of the simulated free-running period from the target period of 22 hours:

$$
C_{\tau_{DD}} = \sum_{i=f,W} \left\langle \left(22 - \Delta_i^P\right)^2 \right\rangle_{DD}.
$$

The seventh term $C_{OSC_{DD}}$ penalises solutions with small ($O(10^{-1})$) free-running amplitudes, bounding the parameter set away from regions where self-sustained rhythmicity can be lost through a supercritical Hopf bifurcation. It also penalises solutions for which: 1) the level of WC-1 protein is very large compared to the level of FRQ protein (some studies, e.g. [11], suggest a low WC-1:FRQ ratio); and 2) the level of FRQ protein is very large compared to that of *frq* mRNA. The term also checks that the peak values and amplitudes of *frq* mRNA and FRQ protein are larger in LD than

in DD. $C_{OSC_{DD}}$ has the form:

$$C_{OSC_{DD}} = \sum_{i=f,F,W} \left(1.5/\langle A_i\rangle_{DD}\right)^2 + \left\langle \left(\frac{y_W\left(\theta_W^P\right) - y_F\left(\theta_F^P\right)}{3y_F\left(\theta_F^P\right)}\right)^2 \right\rangle_{DD} + \left\langle \left(\frac{y_F\left(\theta_F^P\right) - y_f\left(\theta_f^P\right)}{3y_f\left(\theta_f^P\right)}\right)^2 \right\rangle_{DD}$$
$$+ \sum_{i=f,F} \left[\left(\langle A_i\rangle_{DD} / \langle A_i\rangle_{LD}\right)^4 + \left(\langle y_i\left(\theta_i^P\right)\rangle_{DD} / \langle y_i\left(\theta_i^P\right)\rangle_{LD}\right)^4\right].$$

The eighth term $C_{\phi_{DD}}$ measures phase errors in DD, contributing errors of O(1) to C for solutions where FRQ peaks 5 hours after its transcript and oscillates in antiphase to WC-1:

$$C_{\phi_{DD}} = \left\langle \left(\frac{24\left(\theta_F^P - \theta_f^P\right)}{\Delta_f^P} - 5\right)^2 \right\rangle_{DD} + \left\langle \left(\frac{24\left(\theta_F^T - \theta_W^P\right)}{\Delta_f^P}\right)^2 \right\rangle_{DD} + \left\langle \left(\frac{24\left(\theta_F^P - \theta_W^T\right)}{\Delta_f^P}\right)^2 \right\rangle_{DD}.$$

The ninth and final term $C_{PF_{DD}}$ penalises DD solutions for which: 1) the amplitude and peak value of WC-1 oscillations increases exponentially (indicating blow-up of the solution in this variable); and 2) the amplitude of *frq* oscillations decreases exponentially (corresponding to the convergence of *frq* to an equilibrium). $C_{PF_{DD}}$ is given by the expression

$$C_{PF_{DD}} = F_{PF_{DD}}\left([A_W]_{DD}^B, [A_W]_{DD}^E, 0, 0.25\right) + F_{PF_{DD}}\left([A_f]_{DD}^E, [A_f]_{DD}^B, 0, 0.25\right)$$
$$+F_{PF_{DD}}\left(\left[y_W\left(\theta_W^P\right)\right]_{DD}^B, \left[y_W\left(\theta_W^P\right)\right]_{DD}^E, \left[y_W\left(\theta_W^T\right)\right]_{DD}^B, 0.25\right),$$

where:

$$F_{PF_{DD}}\left(V_1, V_2, V_3, E_V\right) = \begin{cases} 0 & ;\ \text{if } \mathrm{V}_1 \geq \mathrm{V}_2 \\ \left(\log\left(E_V\right) / \log\left(\left(V_2 - V_1\right) / \left(V_2 - V_3\right)\right)\right)^2 & ;\ \text{if } \mathrm{V}_1 < \mathrm{V}_2 \end{cases}.$$

Low values of $C_{PF_{DD}}$ favour solutions for which the system exhibits autonomous, bounded oscillations in free-running conditions.

2.2 Description of the optimisation algorithm

To find parameter sets consistent with the target experimental features encoded in the cost function C, we first computed the cost at 50 million quasi-randomly distributed points in the 33-dimensional parameter space of the model. These were generated using a variant of the Sobol algorithm [12] in order to obtain uniform coverage of the space [13]. The points were chosen so that all parameters were bounded between 0 and 10, excluding g, n, h, m, k, f_1, f_2, γ_1, γ_2 and a_4. The Hill coefficients g, n, h, m and k were bounded between 1 and 4 [10]. The upper bound ensured that the search for oscillatory solutions of the free-running system did not arbitrarily increase these parameters and hence bias the resulting solutions towards very high levels of transcription factor/promoter cooperativity. f_1 and f_2 were bounded between 0.1 and 1 so as to keep the mean delays in FRQ and WC-1 production between 2 and 20 hrs. γ_1 and γ_2 were bounded above by $0.05f_1$ and $0.05f_2$ respectively in order to exclude solutions with low protein survival rates. Finally, a_4 was bounded below d_3 to ensure that *wc-1* mRNA stabilised at a constant level in DD (cf. equation (S.3)).

The 50 solutions with the lowest cost function scores were then passed to a variant of the simulated annealing algorithm described in [14]. The annealing schedule employed comprised a million random steps with a linear temperature decrease. The starting temperature for each parameter set

was taken to be the mean cost function score of the 50 best solutions [10]. During the annealing process, the parameter bounds on g, n, h, m, k, f_1, f_2, γ_1, γ_2 and a_4 detailed above were preserved. In addition, transcription and translation rates ($a_1 \rightarrow a_7$) were allowed to vary between 0 and 100, all Michaelis-Menten constants ($b_1 \rightarrow b_{10}$) were allowed to vary between 0 and 25 , and all transport and degradation transport rates (r_1, r_2 and $d_1 \rightarrow d_5$) were allowed to vary between 1 and 200. The 45 annealed solutions with the lowest values of C were then selected for further analysis. The mean and standard deviation of the cost for these parameter sets was 110.72 and 90.67 respectively, while the optimal parameter set used to generate the simulations shown in the Results section had a cost value $C = 95.27$. Throughout the random search and annealing procedures the cost function was capped at a maximum value $C_{MAX} = 10^4$. A total of 5 optimisation runs were carried out, of which the first 4 were test-runs used to fine-tune the cost function and optimisation protocols. Final parameter sets were taken from the outputs of the 5th run.

2.3 Comparison of experimental and *in silico* protein degradation rates

Although the parameters in our model of the *Neurospora* clock are unknown, necessitating the use of numerical optimisation methods, the rates at which the proteins comprising the core clock are degraded in DD have been estimated experimentally [1, 4]. Below, we derive approximations to these key rates for the model, allowing us to compare the experimental values against those obtained from the optimisation procedure.

In [1] and [4], net FRQ and WC-1 protein degradation rates d_{FRQ} and d_{WC-1} were computed from experimental time courses, assuming first order decay with negligible synthesis following an LL to DD transfer. Following [1, 4], the dynamics of total FRQ protein over the time interval of interest is approximated by:

$$F_T(t) = F_T(0)\, e^{-d_{FRQ} t}. \tag{S.17}$$

Differentiating (S.17), substituting into (S.15) and setting $t = 0$ yields:

$$d_{FRQ} = \frac{1}{F_T(0)} \left(\gamma_1 \left(E_1^F(0) + E_2^F(0) \right) + d_2 \frac{P_F(0)}{P_F(0) + b_6} \right). \tag{S.18}$$

At $t = 0$, $\dot{E}_2^F, \dot{P}_F \approx 0$. Equations (S.7)-(S.9) can therefore be used to express both $P_F(0)$ and $E_2^F(0)$ in terms of $E_1^F(0)$. Using the fact that $\gamma_1 \ll f_1$ and $f_1 E_1^F(0) \ll d_2$ then leads to the approximation below:

$$d_{FRQ} = \frac{\gamma_1 \left(2 + \frac{\gamma_1}{f_1} \right) + f_1}{2 + \frac{\gamma_1}{f_1} + \frac{b_6 f_1}{d_2}}. \tag{S.19}$$

A similar argument yields the following approximation to the net WC-1 degradation rate:

$$d_{WC-1} = \frac{\gamma_2 \left(2 + \frac{\gamma_2}{f_2} \right) + f_2}{2 + \frac{\gamma_2}{f_2} + \frac{b_9 f_2}{d_4}}. \tag{S.20}$$

For both FRQ and WC-1, the net degradation rate thus depends on both the delay parameter and the loss rate for intermediate protein species, together with the maximum rate of active protein degradation and the corresponding Michaelis constant. This complex dependence of the rates on a combination of kinetic parameters was observed previously in a temperature-dependent version of the model considered here [2]. Substituting the optimised values of these parameters into (S.19)

and (S.20) gives $d_{FRQ} = 0.12$ and $d_{WC-1} = 0.07$. These compare favourably with the experimental values $d_{FRQ} = 0.27$ ([1]) and $d_{WC-1} = 0.02$ ([3]), being well within an order of magnitude with FRQ degraded faster than WC-1. The subset of model parameters controlling the degradation rates thus lie within biologically reasonable bounds, indicating that they have been appropriately constrained by the optimisation protocol.

3 Quantifying the sensitivity of phase to changes in dawn and dusk

For 24 hr LD cycles, the sensitivities of a circadian phase measure ϕ with respect to changes in the times of dawn t_{DAWN} and dusk t_{DUSK} are determined by the corresponding partial derivatives $\partial\phi/\partial t_{DAWN}$ and $\partial\phi/\partial t_{DUSK}$. If t_{DAWN} and t_{DUSK} are changed by amounts Δt_{DAWN} and Δt_{DUSK}, then to lowest order the resulting change $\Delta\phi$ in ϕ is given by the expression below:

$$\Delta\phi = \frac{\partial\phi}{\partial t_{DAWN}}\Delta t_{DAWN} + \frac{\partial\phi}{\partial t_{DUSK}}\Delta t_{DUSK}. \tag{S.21}$$

Assuming the clock is stably entrained, perturbing t_{DAWN} and t_{DUSK} simultaneously by the same amount Δt will result in ϕ changing by Δt also. It therefore follows from (S.21) that $\partial\phi/\partial t_{DAWN}$ and $\partial\phi/\partial t_{DUSK}$ always sum to 1. The comparative size of the derivatives thus indicates the relative response of ϕ to changes in dawn and dusk. In particular, a dawn sensitivity $\partial\phi/\partial t_{DAWN}$ equal to 1 means that the phase is locked to dawn since the resulting change in ϕ will be equal to Δt_{DAWN} (and independent of Δt_{DUSK}). In light response plots such as Figures 4A and 6A of the main paper, this corresponds to the line showing the change in ϕ with photoperiod lying exactly parallel to the line indicating the corresponding change in the time of dawn. A dusk sensitivity $\partial\phi/\partial t_{DUSK}$ of 1 implies that the phase is perfectly locked to dusk; in this case ϕ lies parallel to the line indicating the time of dusk.

Since the value of $\partial\phi/\partial t_{DAWN}$ determines that of $\partial\phi/\partial t_{DUSK}$ and vice versa, only one of these is necessary as a measure of the degree of dawn/dusk dominance. Here, we use the dusk sensitivity $\partial\phi/\partial t_{DUSK}$. Values of $\partial\phi/\partial t_{DUSK}$ close to 0 and 1 represent dawn- and dusk-locking respectively, while intermediate values indicate a systematic change in ϕ with photoperiod (ϕ non-parallel to both dawn and dusk). A value of 0.5 denotes exactly equal dawn and dusk sensitivities. For Figures 4A and 6A where photoperiod is varied through equal and opposite changes to dawn and dusk, this corresponds to a net phase change $\Delta\phi$ of zero. This can be seen by substituting $\partial\phi/\partial t_{DAWN} = \partial\phi/\partial t_{DUSK} = 0.5$ and $\Delta t_{DAWN} = -\Delta t_{DUSK}$ into equation (S.21). A zero phase change for such light-forcing protocols denotes a clock that tracks the middle of the night, modulo a fixed phase shift.

Driven and systematic entrainment can also be quantified using the sensitivity of phase with respect to photoperiod, $\partial\phi/\partial P$. Applying the chain rule and using the relations $t_{DAWN} = -t_{DUSK} = P/2$ and $\partial\phi/\partial t_{DAWN} + \partial\phi/\partial t_{DUSK} = 1$ yields:

$$\frac{\partial\phi}{\partial P} = \frac{1}{2}\left(2\frac{\partial\phi}{\partial t_{DUSK}} - 1\right). \tag{S.22}$$

(S.22) shows that $\partial\phi/\partial P$ is bounded between -0.5 and 0.5, with these values indicating dawn- and dusk-locking respectively. Midnight-tracking corresponds to a $\partial\phi/\partial P$ value of 0, as can be seen by setting $\partial\phi/\partial t_{DUSK} = 0.5$.

For the symmetric T-cycles considered in Figure 5 of the main paper, t_{DAWN} and t_{DUSK} are functions of T, with $t_{DAWN} = -t_{DUSK} = T/4$. Consequently, dawn- and dusk-locking correspond to phase derivatives $d\phi/dT$ equal to 1/4 and -1/4 respectively; a derivative of 0 indicates a system that responds equally to both transitions. In this case, the following linear function of the phase derivative provides a suitable dusk sensitivity index:

$$\frac{\partial \phi}{\partial t_{DUSK}} \overset{def}{=} \frac{1}{2}\left(1 - 4\frac{d\phi}{dT}\right).$$

By construction, $\partial\phi/\partial t_{DUSK}$ is bounded between 0 (dawn-locking) and 1 (dusk-locking), with a value of 0.5 characterising a clock that tracks midnight.

It should be noted that the sensitivity measures introduced in this section are unaffected by any parameter transformations that preserve the corresponding phase measures. In particular, they are invariant under parameter changes $k = (k_1, \ldots, k_s) \mapsto Rk = (R_1 k_1, \ldots, R_s k_s)$ that result in the components $\gamma_i(t, k)$ of the periodic solution $\gamma(t, k)$ of the entrained model being rescaled (i.e. for which $\gamma_i(t, Rk) = S_i \gamma_i(t, k)$ with $S_i > 0$)). Thus, although our emphasis in this work was to reproduce key qualitative circadian properties preserved across data sets, the sensitivity results presented here would not be altered by any rescaling of the model time series to match a particular set of experimental expression levels.

4 Measuring the robustness of phase to changes in photoperiod

Given a fixed parameter set $k = (k_1, \ldots, k_s)$, the robustness of phase with respect to changes in photoperiod P over a range $P_1 \leq P \leq P_2$ was measured using the quantity

$$R_{\phi_{FRQ}}(k) = \frac{1}{P_2 - P_1}\int_{P_1}^{P_2} D_{\phi_{FRQ}}(k, P)\, dP, \tag{S.23}$$

where the evaluation function $D_{\phi_{FRQ}}(k, P)$ is defined by:

$$D_{\phi_{FRQ}}(k, P) = 4\left(0.5^2 - \frac{\partial \phi_{FRQ}}{\partial P}(k, P)^2\right). \tag{S.24}$$

Recall from section 3 that the sensitivity of entrained phase to photoperiod $\partial\phi_{FRQ}(k, P)/\partial P$ is bounded between -0.5 and 0.5. A sensitivity of 0.5 indicates a clock that is locally dusk-driven (i.e. is locked to dusk in in an interval around P); -0.5 a clock that is locally dawn-driven; and 0 a system that is locally systematically entrained (tracks midnight in an interval around P). It therefore follows from the form of (S.24) that $0 \leq D_{\phi_{FRQ}}(k, P) \leq 1$, with $D_{\phi_{FRQ}}(k, P) = 0$ and $D_{\phi_{FRQ}}(k, P) = 1$ denoting local driven and systematic entrainment respectively. It follows in turn that $0 \leq R_{\phi_{FRQ}}(k) \leq 1$, with minimum robustness indicating global driven entrainment (dawn- or dusk tracking over the entire interval (P_1, P_2)) and maximum robustness indicating global systematic entrainment (midnight-tracking over (P_1, P_2)).

The measure $R_{\phi_{FRQ}}(k)$ can thus be used to quantify the effect of structural changes to the clock circuitry on the robustness of entrained phase with respect to photoperiod. Let k_{WT} and k represent the parameters of the WT and modified systems respectively. Then the ratio below provides a suitable measure of the change in robustness:

$$R_{\phi_{FRQ}}(k|k_{WT}) = \frac{R_{\phi_{FRQ}}(k)}{R_{\phi_{FRQ}}(k_{WT})}.$$

A $R_{\phi_{FRQ}}(k|k_{WT})$ value greater than 1 implies a clock that is more robust than the WT; a value less than 1 implies a system less robust than the WT.

Here, we were interested in the effect of uncoupling the positive *wc-1* loop from the central *frq* loop. Since the coupling strength is determined by the parameter a_7, the measure

$$R_{\phi_{FRQ}}\left(a_7|a_7^{WT}\right) = \frac{R_{\phi_{FRQ}}(a_7)}{R_{\phi_{FRQ}}\left(a_7^{WT}\right)},$$

quantifies how the robustness of entrained phase against photoperiod fluctuations varies with the strength of positive feedback. (In the above, $R_{\phi_{FRQ}}(a_7)$ denotes phase robustness calculated using (S.23) and (S.24) for a feedback strength a_7, with all other kinetic parameters fixed at their WT values). For all computations reported in this work, we chose minimum and maximum photoperiods of $P_1 = 6$ hrs and $P_2 = 18$ hrs respectively

To conclude this section, we recall from the discussion at the end of section 3 that the sensitivity measure $\partial\phi_{FRQ}(k,P)/\partial P$ is invariant under parameter changes $k \mapsto Rk$ which rescale the limit cycle; that is $\partial\phi_{FRQ}(Rk,P)/\partial P = \partial\phi_{FRQ}(k,P)/\partial P$. It therefore follows that $D_{\phi_{FRQ}}(Rk,P) = D_{\phi_{FRQ}}(k,P)$ and hence $R_{\phi_{FRQ}}(Rk) = R_{\phi_{FRQ}}(k)$ (cf. equations (S.23) and (S.24)). Phase robustness is thus also invariant to amplitude rescaling. In particular, any such rescaling will preserve the observed variation in robustness with positive feedback strength.

5 Quantifying network flexibility

5.1 Measuring the flexibility of circadian outputs

The flexibility measure used in this study is based on analysing the map relating parameter variations to changes in key circadian outputs. Following [15, 16], we consider the effect of varying the parameters $k = (k_1, \ldots, k_s)$ of the model on clock outputs Q_j computable from the periodic solution $\gamma(t,k)$ of the model corresponding to the entrained clock (i.e. the limit cycle attractor of the system). Reasonable outputs Q_j within a circadian context here include quantities such as entrained phase, the phases of the minima and maxima of mRNA and protein profiles, the amplitude of these minima and maxima, and - when considering the global flexibility of the system - the entrained limit cycle γ itself [15, 16]. When the parameters are changed (usually by small amounts) then the variation is denoted by $\delta k = (\delta k_1, \ldots, \delta k_s)$. Each variation of the parameters from k to $k + \delta k$ will cause the limit cycle to vary, and this in turn changes the vector of outputs $Q = (Q_1, \ldots, Q_s)$ by an amount $\delta Q = (\delta Q_j)$. The variation δk is an absolute one in that the size of each change δk_i is independent of the size of k_i. However, since the k_is can vary over more than one order of magnitude (see Table 1 of the main paper), it is more appropriate to consider the proportional variation in k_i given by the quantity $\delta\eta_i = \delta k_i/k_i$ (the $\delta\eta_i$s also have the advantage of being dimensionless). If the scaled variations $\delta\eta_i$ are small, then the map $\delta k \mapsto \delta Q$ relating parameter and output changes can be approximated by its linearisation M, the $m \times s$ matrix whose components are the partial derivatives of the individual outputs with respect to the parameters $M_{ij} = \frac{\partial Q_i}{\partial k_j}(k)$. In terms of the scaled parameter changes $\delta\eta = (\delta\eta_i)$, the change δQ in Q is given by

$$\delta Q = M^*\delta\eta, \qquad \text{(S.25)}$$

where $M^* = M\Delta_k$ is the product of M with the $s \times s$ diagonal matrix $\Delta_k = diag(k_1, \ldots, k_s)$ (the elements of M^* are thus the scaled partial derivatives, $M^*_{ij} = k_j \frac{\partial Q_i}{\partial k_j}(k)$). The changes to the outputs of the system resulting from small random parameter perturbations can therefore be understood by analysing the form of M^*. An important tool in this analysis is the singular value decomposition (SVD) of M^* [15, 16]. The SVD of M^* is its factorisation into the form

$$M^* = U\Sigma V^T, \tag{S.26}$$

where U is an $m \times s$ column-orthonormal matrix ($U^T U = I_s$), V is an $s \times s$ orthonormal matrix ($V^T V = V V^T = I_s$) and $\Sigma = diag(\sigma_1, \ldots, \sigma_s)$ is an $s \times s$ diagonal matrix. The elements $\sigma_1 \geq \cdots \geq \sigma_s \geq 0$ are the singular values of M^* while the columns u_i of U and v_j of V are the left and right singular vectors of M^* respectively [13].

Following [15,16], we consider the changes to the output vector $\delta Q^{(\ell)}$ arising from variations $\delta\eta_i^{(\ell)}$ ($\ell = 1, 2, \ldots, N$) in the scaled parameters, where the $\delta\eta_i^{(\ell)}$s are taken to be zero-mean independent identically distributed random variables with variance $\left\langle \left(\delta\eta_i^{(\ell)}\right)^2 \right\rangle = r_\eta^2$ (angular brackets here denote the expectation of a random variable). As $N \longrightarrow \infty$, this ensemble can be thought of as the set of all possible parameter fluctuations that can result from stochastic evolutionary processes of a bounded size. Provided that r_η is not too large, (S.25) implies that $\delta Q^{(\ell)}$ is approximated by $M^* \delta\eta^{(\ell)}$. It can then be shown that in the limit $N \longrightarrow \infty$, the principal components of the ensemble $\left\{\delta Q^{(\ell)}\right\}$ are the pairs $\left\{u_i, r_\eta^2 \sigma_i^2\right\}$ of M^* [16]. The left singular vectors $\{u_1, \ldots, u_s\}$ of M^* thus provide an orthogonal coordinate system within which the $\delta Q_i^{(\ell)}$s are uncorrelated, with variances equal to $r_\eta^2 \sigma_i^2$. Geometrically, the ensemble $\left\{\delta\eta^{(\ell)}\right\}$ can be thought of as a ball of parameter perturbations that are mapped to an ellipsoid of output perturbations $\left\{\delta Q^{(\ell)}\right\}$ by M^*; u_i and $r_\eta \sigma_i$ are then the ith principal axis of the ellipse and the extent of the ellipse along this axis respectively. The singular values σ_i thus quantify the effect of random parameter perturbations on the output Q. Furthermore, a scalar measure of the size of the output variations is provided by the net variance $r_\eta^2 \sum_{i=1}^s \sigma_i^2$ (it is straightforward to show that this is equal to the average of the squared output perturbation size, $\left\langle \left\|\delta Q^{(\ell)}\right\|^2 \right\rangle$). The sum of the singular values $\sum_{i=1}^s \sigma_i^2$ therefore provides a simple measure of the flexibility of Q: the larger this sum, the greater the relative change in Q under random parameter perturbations. In addition, the left singular vectors u_i associated with the largest singular values indicate the most flexible (or evolutionarily accessible) directions in the output space. These are the directions along which relatively large changes in Q can be obtained with comparatively small changes $\delta\eta$ in the parameters. Geometrically, these correspond to the principal axes of the ellipsoid of output perturbations along which it has greatest extent [15, 16]. The corresponding right singular vectors v_i represent the parameter changes that lead to variations along the principal axes of the ellipsoid: i.e. those parameter variations most likely to be achieved by evolutionary processes.

5.2 Calculating the relative flexibility of the clock network

In order to quantify the effect on circadian flexibility of perturbations to the wild-type parameter set k_{WT}, we consider a relative flexibility measure given by the expression below:

$$F_Q(k|k_{WT}) = \frac{\sum_{i=1}^s \sigma_i(k)^2}{\sum_{i=1}^s \sigma_i(k_{WT})^2}.$$

Here, $\sigma_i(k_{WT})$ and $\sigma_i(k)$ are the singular values for the WT and modified system respectively. If $F_Q(k|k_{WT})$ is greater than 1, Q is more flexible in the modified system than in the WT, while if it is less than 1 it is less flexible.

For the *Neurospora* model, we were interested in the effect of reducing the strength of FRQ's positive feedback on WC-1 production. As all circadian outputs of interest can be computed from the limit cycle attractor γ of the entrained system, a suitable global flexibility index for this analysis is provided by:

$$F_\gamma\left(a_7|a_7^{WT}\right) = \frac{\sum_{i=1}^{s}\sigma_i(a_7)^2}{\sum_{i=1}^{s}\sigma_i\left(a_7^{WT}\right)^2}. \tag{S.27}$$

For a given positive feedback strength a_7, $F_\gamma\left(a_7|a_7^{WT}\right)$ compares the variance of the corresponding limit cycle under random parameter perturbations to the variance for WT coupling; values less than 1 indicate a system that is less flexible than the WT.

In (S.27), $\sigma_i(a_7)$ is the ith singular value of the matrix M^* relating parameter perturbations $\delta\eta$ to the corresponding change δQ to the vector of system outputs Q in the case where Q is the entrained limit cycle γ (cf. equation (S.25)). Writing the ith component of $\gamma(t,k)$ as $\gamma_i(t,k)$, Q is therefore the infinite-dimensional vector

$$Q = (\gamma_1(0,k),\ldots,\gamma_1(T,k),\gamma_2(0,k),\ldots,\gamma_2(T,k)\ldots,\gamma_4(0,k),\ldots,\gamma_4(T,k))^T, \tag{S.28}$$

where T is the period of the forcing light-dark cycle and $\gamma_1 \longrightarrow \gamma_4$ are *frq* mRNA, *wc-1* mRNA, total FRQ protein and total WC-1 protein concentrations respectively. The matrix $M^* = \left(k_j \frac{\partial\gamma_i}{\partial k_j}(t,k)\right)$ is thus the linearisation of a map from scaled parameter variations $\delta\eta \in \mathbb{R}^s$ to the space of T-periodic, 4-dimensional, smoothly differentiable real-valued functions [15, 16]. Following [15, 16], singular values and vectors were calculated from a finite approximation to M^* obtained by restricting time t to a discrete set of $N \gg 1$ evenly spaced values in the interval $[0,T]$.

Finally, it is straightforward to show that normalising the derivative $\frac{\partial\gamma_i}{\partial k_j}(t,k)$ in M^* by the magnitude of the limit cycle $\|\gamma_i(t,k)\|$ preserves the SVD under the amplitude rescaling parameter transformations discussed in sections 3 and 4.[1] It follows that the corresponding flexibility measure $\hat{F}_\gamma$ is also invariant under any rescaling of this type. We found that the variation of $\hat{F}_\gamma$ with positive feedback strength a_7 is very similar to that observed for the non-normalised measure F_γ. Thus - despite the greater generality of the normalised measure - non-normalised flexibility is plotted in Figure 7 of the main paper for simplicity.

6 Phase and amplitude variations for the entrained limit cycle

The limit cycle $\gamma_i(t,k)$ resulting from a general parameter perturbation $k_0 \mapsto k$ can be approximated by the combination of a phase change $s_i(k)$ and a relative amplitude change $A_i(k)$:

$$\gamma_i(t,k) = (1 + A_i(k))\gamma_i(t + s_i(k), k_0). \tag{S.29}$$

[1]Here, $\|.\|$ is the norm induced by the inner product $\langle p(t), q(t)\rangle = \frac{1}{T}\int_0^T p(s)\,q(s)\,ds$, so that $\|p(t)\| = \sqrt{\langle p(t), p(t)\rangle} = \sqrt{\frac{1}{T}\int_0^T p(s)^2\,ds}$.

A perturbation of magnitude α in a particular direction w results, to lowest order, in the phase and amplitude variations

$$s_i(k) = \alpha \frac{\partial s_i}{\partial w}(k_0), \tag{S.30}$$

$$A_i(k) = \alpha \frac{\partial A_i}{\partial w}(k_0), \tag{S.31}$$

where $\partial s_i(k_0)/\partial w$ and $\partial A_i(k_0)/\partial w$ denote the directional derivatives of $A_i(k)$ and $S_i(k)$ along w evaluated at k_0. Here, we derive analytical expressions for these derivatives in terms of the principal components of γ. We also show that in the case when the first principal component u_1 is proportional to the derivative of the limit cycle, that is $u_1 = \beta\dot{\gamma}$, the derivatives along the principal parameter direction v_1 take the values $\partial s_i(k_0)/\partial v_1 = \beta\sigma_1$ and $\partial A_i(k_0)/\partial v_1 = 0$. This implies that $s_i(k) \approx \alpha\beta\sigma_1$ and $A_i(k) \approx 0$, corresponding to a uniform phase change (cf. equations (S.29)-(S.31)).

Write $k_0 = (k_{01}, \ldots, k_{0s})$. Since we consider proportional parameter changes here, $\partial s_i(k_0)/\partial w$ and $\partial A_i(k_0)/\partial w$ are given by the expressions below:

$$\frac{\partial s_i}{\partial w}(k_0) = \left(k_{01}\frac{\partial s_i}{\partial k_1}(k_0), \ldots, k_{0s}\frac{\partial s_i}{\partial k_s}(k_0)\right) \cdot w, \tag{S.32}$$

$$\frac{\partial A_i}{\partial w}(k_0) = \left(k_{01}\frac{\partial A_i}{\partial k_1}(k_0), \ldots, k_{0s}\frac{\partial A_i}{\partial k_s}(k_0)\right) \cdot w. \tag{S.33}$$

Differentiating both sides of (S.29) with respect to k_j implies the following equation for $\partial\gamma_i(t,k_0)/\partial k_j$:

$$\frac{\partial \gamma_i}{\partial k_j}(t,k_0) = \frac{\partial s_i}{\partial k_j}(k_0)\dot{\gamma}_i(t,k_0) + \frac{\partial A_i}{\partial k_j}(k_0)\gamma_i(t,k_0).$$

Using the identity $\int_0^T \gamma_i(s,k)\dot{\gamma}_i(s,k)\,ds = 0$ then yields

$$k_{0j}\frac{\partial s_i}{\partial k_j}(k_0) = \frac{\left\langle k_{0j}\frac{\partial \gamma_i}{\partial k_j}(t,k_0), \dot{\gamma}_i(t,k_0)\right\rangle}{\|\dot{\gamma}_i(t,k_0)\|^2} \tag{S.34}$$

and

$$k_{0j}\frac{\partial A_i}{\partial k_j}(k_0) = \frac{\left\langle k_{0j}\frac{\partial \gamma_i}{\partial k_j}(t,k_0), \gamma_i(t,k_0)\right\rangle}{\|\gamma_i(t,k_0)\|^2}. \tag{S.35}$$

The terms $k_{0j}\partial\gamma_i(t,k_0)/\partial k_j$ in (S.34) and (S.35) are the elements of the matrix M^*. It therefore follows from the SVD expansion (S.26) of M^* that

$$k_{0j}\frac{\partial s_i}{\partial k_j}(t,k_0) = \frac{\sum_{l=1}^{s}\sigma_l v_{jl}\left\langle u_l^i(t,k_0), \dot{\gamma}_i(t,k_0)\right\rangle}{\|\dot{\gamma}_i(t,k_0)\|^2} \tag{S.36}$$

and

$$k_{0j}\frac{\partial A_i}{\partial k_j}(t,k_0) = \frac{\sum_{l=1}^{s}\sigma_l v_{jl}\left\langle u_l^i(t,k_0), \gamma_i(t,k_0)\right\rangle}{\|\gamma_i(t,k_0)\|^2}, \tag{S.37}$$

where v_{jl} is the (j,l)th element of the matrix V and $u_l^i(t,k_0)$ represents the element of the vector u_l occupying the same position as $\gamma_i(t,k_0)$ in the representation of the limit cycle as an infinite-dimensional vector (cf. equation (S.28)). Substituting (S.36) and (S.37) into (S.32) and (S.33)

respectively and using the relation $V^T V = I_s$ leads to the final expressions for $\partial s_i(k_0)/\partial w$ and $\partial A_i(k_0)/\partial w$ below:

$$\frac{\partial s_i}{\partial w}(k_0) = \frac{\sum_{l=1}^{s}(v_l \cdot w)\,\sigma_l\,\langle u_l^i(t,k_0),\dot{\gamma}_i(t,k_0)\rangle}{\|\dot{\gamma}_i(t,k_0)\|^2}, \tag{S.38}$$

$$\frac{\partial A_i}{\partial w}(k_0) = \frac{\sum_{l=1}^{s}(v_l \cdot w)\,\sigma_l\,\langle u_l^i(t,k_0),\gamma_i(t,k_0)\rangle}{\|\gamma_i(t,k_0)\|^2}. \tag{S.39}$$

The phase derivative is thus obtained by projecting the principal components of the limit cycle onto the derivative of the cycle while the amplitude derivative is obtained by projection onto the cycle itself.

In the case when the first principal component is proportional to the derivative, so that $u_1^i(t,k_0) = \beta\dot{\gamma}_i(t,k_0)$, setting $w = v_1$ in (S.38) and (S.39) implies

$$\frac{\partial s_i}{\partial v_1}(k_0) = \frac{\beta\sigma_1\,\langle\dot{\gamma}_i(t,k_0),\dot{\gamma}_i(t,k_0)\rangle}{\|\dot{\gamma}_i(t,k_0)\|^2} = \beta\sigma_1$$

since $\langle\dot{\gamma}_i(t,k_0),\dot{\gamma}_i(t,k_0)\rangle = \|\dot{\gamma}_i(t,k_0)\|^2$, and

$$\frac{\partial A_i}{\partial v_1}(k_0) = \frac{\beta\sigma_1\,\langle\dot{\gamma}_i(t,k_0),\gamma_i(t,k_0)\rangle}{\|\gamma_i(t,k_0)\|^2} = 0$$

since $\langle\dot{\gamma}_i(t,k_0),\gamma_i(t,k_0)\rangle = 0$. The perturbation along v_1 thus results in a uniform phase change, as claimed.

We conclude by remarking that in common with the phase sensitivity and robustness measures discussed in sections 3 and 4, $\partial s_i(k_0)/\partial w$ and $\partial A_i(k_0)/\partial w$ are also invariant under parameter transformations that rescale amplitude, $\gamma_i(t,k_0) \mapsto S_i\gamma_i(t,k_0)$. This is a consequence of the fact that in addition to scaling $\gamma_i(t,k_0)$ (and hence $\dot{\gamma}_i(t,k_0)$) by S_i, such a transformation will scale the elements $u_l^i(t,k_0)$ of U in the factorisation $M^* = U\Sigma V^T$ by S_i also. The invariance of the derivatives then follows directly from the forms of (S.38) and (S.39).

References

[1] Ruoff P, Loros JJ, Dunlap JC: **The relationship between FRQ-protein stability and temperature compensation in the Neurospora circadian clock**. *Proc Natl Acad Sci USA* 2005, **102**(49):17681–6.

[2] Akman OE, Locke JCW, Tang S, Carré I, Millar AJ, Rand DA: **Isoform switching facilitates period control in the Neurospora crassa circadian clock.** *Mol Sys Biol* 2008, **4:64**.

[3] Hong CI, Jolma IW, Loros JJ, Dunlap JC, Ruoff P: **Simulating dark expressions and interactions of frq and wc-1 in the Neurospora circadian clock.** *Biophys J* 2008, **94**(4):1221–32.

[4] Hong CI, Ruoff P, Loros JJ, Dunlap JC: **Closing the circadian negative feedback loop: FRQ-dependent clearance of WC-1 from the nucleus.** *Genes Dev* 2008, **22**(22):3196–3204.

[5] Froehlich AC, Liu Y, Loros JJ, Dunlap JC: **White collar-1, a circadian blue light photoreceptor, binding to the frequency promoter.** *Science* 2002, **297**(5582):815–9.

[6] Crosthwaite SK, Loros JJ, Dunlap JC: **Light-induced resetting of a circadian clock is mediated by a rapid increase in frequency transcript.** *Cell* 1995, **81**(7):1003–12.

[7] Tan Y, Dragovic Z, Roenneberg T, Merrow M: **Entrainment dissociates transcription and translation of a circadian clock gene in Neurospora.** *Curr Biol* 2004, **14**(5):433–8.

[8] Linden H, Macino G: **White collar 2, a partner in blue-light signal transduction, controlling expression of light-regulated genes in Neurospora crassa.** *EMBO J* 1997, **16**:98–109.

[9] MacDonald N: *Biological Delay Systems: Linear Stability Theory.* Cambridge University Press; 1989.

[10] Locke JCW, Millar AJ, Turner MS: **Modelling genetic networks with noisy and varied experimental data: the circadian clock in Arabidopsis thaliana.** *J Theor Biol* 2005, **234**(3):383–93.

[11] Schafmeier T, Haase A, Káldi K, Scholz J, Fuchs M, Brunner M: **Transcriptional feedback of Neurospora circadian clock gene by phosphorylation-dependent inactivation of its transcription factor.** *Cell* 2005, **122**(2):235–46.

[12] Bratley P, Fox BL: **ALGORITHM 659: Implementing Sobol's quasirandom sequence generator**. *ACM Trans Math Softw* 1988, **14**:88–100.

[13] Press WH, Teukolsky SA, Vetterling WT, Flannery BP: *Numerical Recipes in C: The Art of Scientific Computing.* Cambridge University Press; 1996.

[14] Kirkpatrick S, Gelatt CD, Vecchi MP: **Optimization by simulated annealing.** *Science* 1983, **220**:671–680.

[15] Rand DA, Shulgin BV, Salazar D, Millar AJ: **Design principles underlying circadian clocks.** *J R Soc Interface* 2004, **1**:119–130.

[16] Rand DA, Shulgin BV, Salazar JD, Millar AJ: **Uncovering the design principles of circadian clocks: mathematical analysis of flexibility and evolutionary goals**. *J Theor Biol* 2006, **238**(3):616–635.

Supplementary Figures

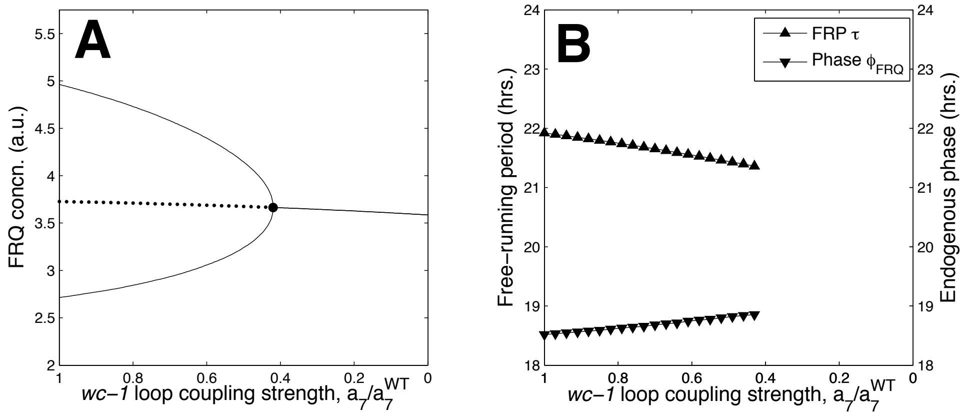

Figure S1: **A**. Bifurcation diagram showing the loss of rhythmicity in the simulated free-running (DD) system as the relative coupling strength a_7/a_7^{WT} of the *wc-1* loop is reduced (the parameter a_7 determines the rate at which FRQ protein upregulates WC-1 production, with a_7^{WT} indicating the WT value). For each coupling strength value on the x-axis, the corresponding values on the y-axis denote minimum and maximum FRQ levels. Solid lines denote stable attractors and broken lines unstable attractors. The solid circle indicates a supercritical Hopf bifurcation at which the attractor changes from a limit cycle (corresponding to rhythmicity) to a fixed point (corresponding to arrhythmicity). **B.** Corresponding variations in free-running period τ and the falling phase ϕ_{FRQ} of FRQ protein. ϕ_{FRQ} is calculated relative to the FRQ minimum, taken as CT 0.

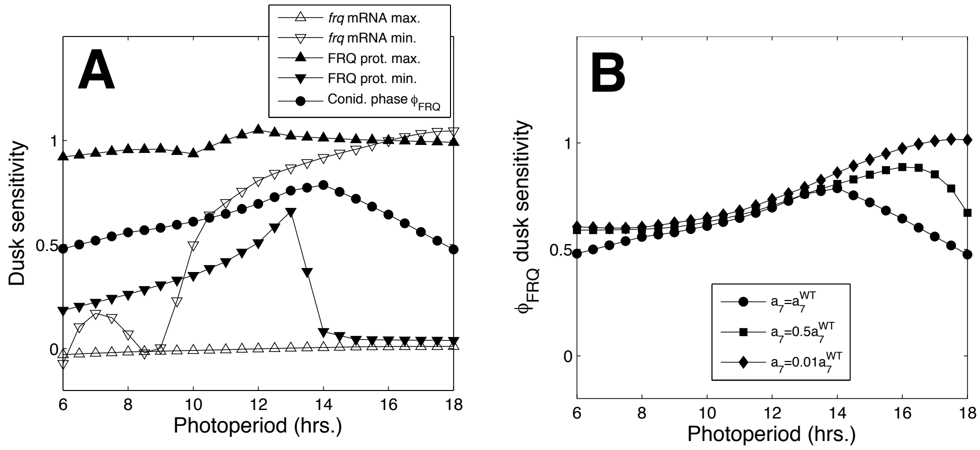

Figure S2: **A**. Variations in dusk sensitivity with photoperiod P for the phase measures plotted in Figure 4 of the main paper. Conidiation phase ϕ_{FRQ} has an intermediate sensitivity across the photoperiod range, indicating systematic entrainment (the higher sensitivities observed close to $P = 14$ correspond to the inflexion of the phase-photoperiod profile in Figure 4A). **B**. The effect of removing the *wc-1* loop on the dusk sensitivity-photoperiod profile of ϕ_{FRQ}. Note the pronounced increase in sensitivity for larger P values as the relative coupling strength a_7/a_7^{WT} is decreased.

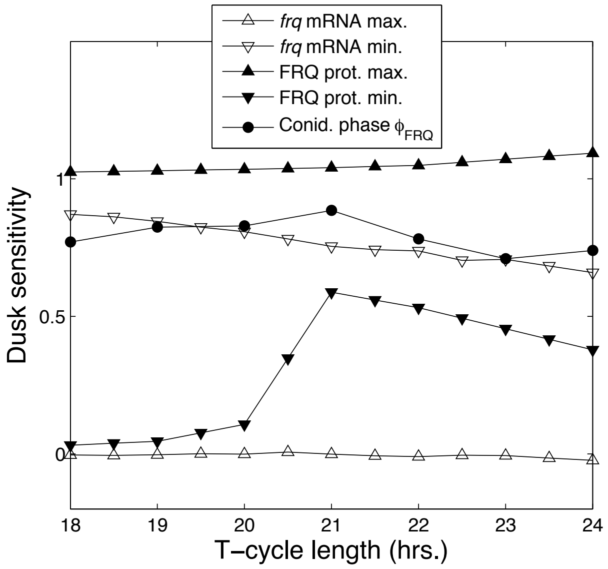

Figure S3: Dependence of the dusk sensitivities plotted in Figure 5 on T-cycle length. Conidiation phase ϕ_{FRQ} has a high sensitivity across the range shown, indicating a dusk-driven response.

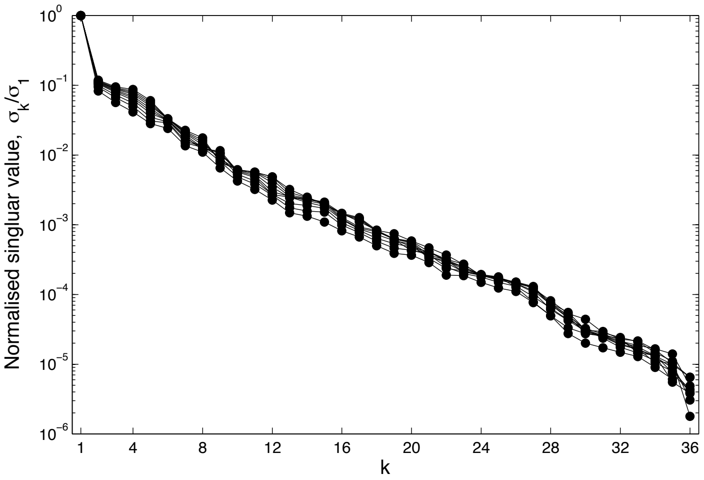

Figure S4: Normalised singular value spectra $\{\sigma_k/\sigma_1\}$ corresponding to each of the relative flexibility computations $F_\gamma\left(a_7|a_7^{WT}\right)$ plotted in Figure 7A. For all *wc-1* loop coupling strengths, the leading singular value σ_1 is larger than the others by an order of magnitude, showing that the decrease in flexibility with coupling occurs primarily along the first principal component of the entrained limit cycle.

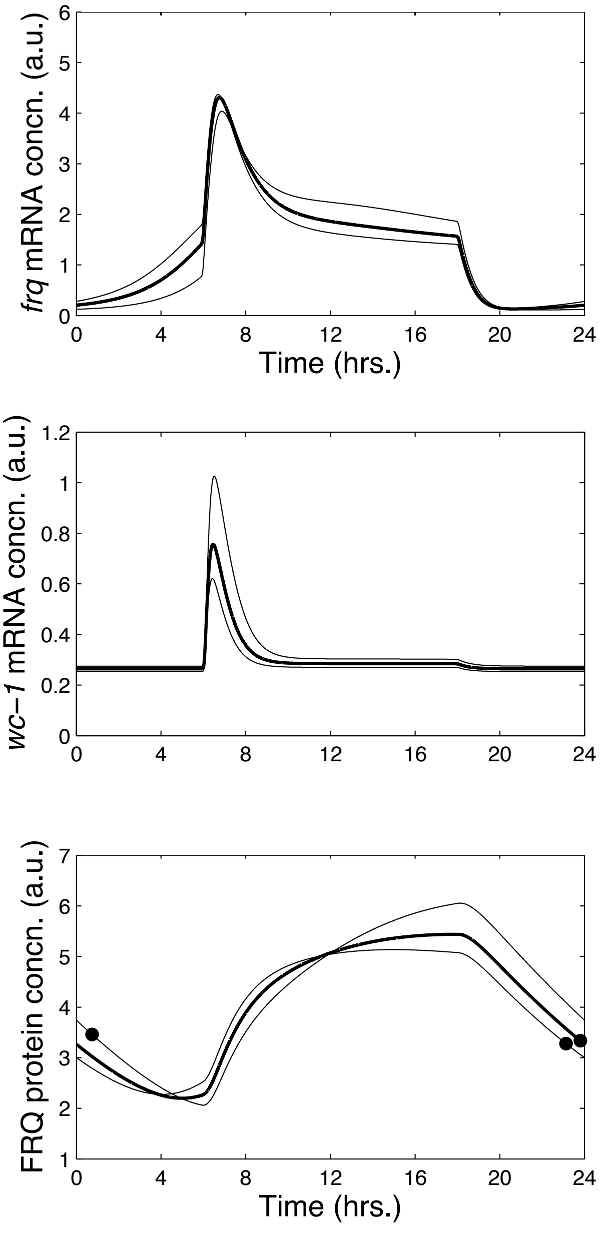

Figure S5: The effect of perturbing the WT solution of the model (thick solid lines) in the direction of the first principle component vector u_1 of the entrained limit cycle γ. Perturbed solutions (thin solid lines) were computed for proportional parameter variations of $\pm 2\%$ (that is for parameters $k_i = k_i^{WT}(1 \pm 0.02 v_{i1})$, where k_i^{WT} are the WT parameters and $v_1 = (v_{i1})$ is the right singular vector associated with u_1). Solid circles represent FRQ-dependent conidiation phase ϕ_{FRQ}. Note the greater flexibility in the phase of FRQ protein compared to that of *frq* and *wc-1* mRNA, consistent with the phase-amplitude sensitivity analysis shown in Figure 8A.

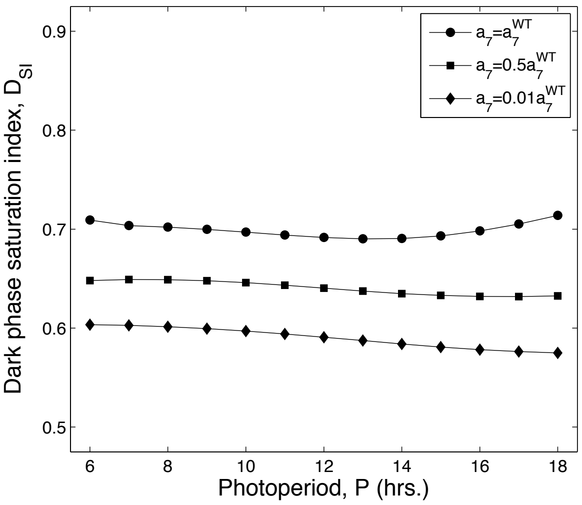

Figure S6: The effect of decreasing positive feedback strength a_7 on the FRQ degradation saturation index D_{SI} for different photoperiods P. D_{SI} is defined by $D_{SI} = \frac{1}{24-P}\int_{t_{DUSK}}^{t_{DUSK}+24-P} \frac{P_F(t)}{P_F(t)+b_6}dt$. Values of the measure closer to 1 denote a near-constant rate of FRQ loss during the night, resulting in FRQ profiles that decrease linearly with time. Note that reducing a_7 uniformly decreases D_{SI} across photoperiods, providing the system with a simple mechanism for tuning the level of saturation.
